# Supplementary material for: The dysregulated Pink1- Drosophila mitochondrial proteome is partially corrected with exercise
Source: Aging (Albany NY). 2021 Jun 1;13(11):14709–28. doi: 10.18632/aging.203128 (PMC8221352; doi:10.18632/aging.203128)

SUPPLEMENTARY MATERIALS

Ingram counter-balanced exerciser

Our bespoke Ingram Counter-balanced Exercise (ICE) machine was adapted from the PT design of Piazza et al., by Mr. John Ingram (TI's father) [82]. The exerciser fits into an incubator with internal dimensions of 48 x 48 x 35cm and provides a vertically moving tray measuring 30 x 34cm. A solenoid-powered counterbalanced lever causes the tray to be lifted 3cm. The tray is lifted and immediately dropped every 15 seconds.

Counterbalancing the lever are a series of springs, which can be adjusted to allow lifts of up to 3.0kg. Springs efficiently store and release energy enabling a more rapid drop than would be the case if weights were used. They also reduce the size and overall weight of the device.

Lift is provided by a solenoid wound onto a nylon core and fixed to the body of the exerciser. The steel armature passing through the solenoid is attached to the counterbalanced lever. Activation of the solenoid causes the armature to rise which lifts the lever.

Solenoid activation is controlled by an astable timer. This triggers a relay to pulse the applied AC voltage. Two capacitors acting as a loss-less resistor allow the voltage to be reduced without producing excess heat, before it is rectified, smoothed and finally applied to the solenoid. Powering with DC current causes less vibration and heat generation in the solenoid but, as the capacitors and the solenoids both work more efficiently at lower temperatures, any excess heat is subsequently dissipated by proximate fans. Using a pulse of current as described rather than the discharge from a large capacitor to activate the solenoid saves space, it is also safer as there is far less stored energy.

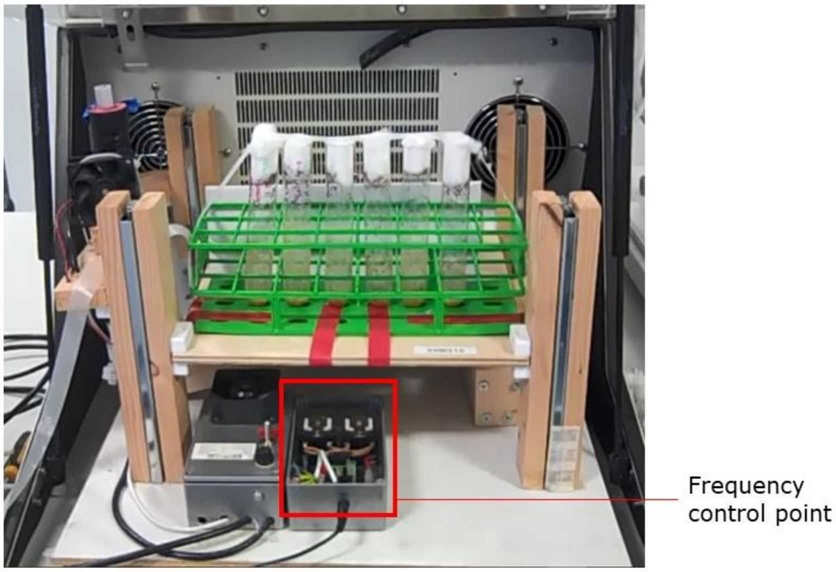

Supplement: Supplementary Materials [file aging-13-203128-s001.pdf]
