# Supplementary material for: The dysregulated Pink1- Drosophila mitochondrial proteome is partially corrected with exercise
Source: Aging (Albany NY). 2021 Jun 1;13(11):14709–28. doi: 10.18632/aging.203128 (PMC8221352; doi:10.18632/aging.203128)
Supplement: Supplementary Figure 1 [file aging-13-203128-s002.pdf]

## SUPPLEMENTARY FIGURE

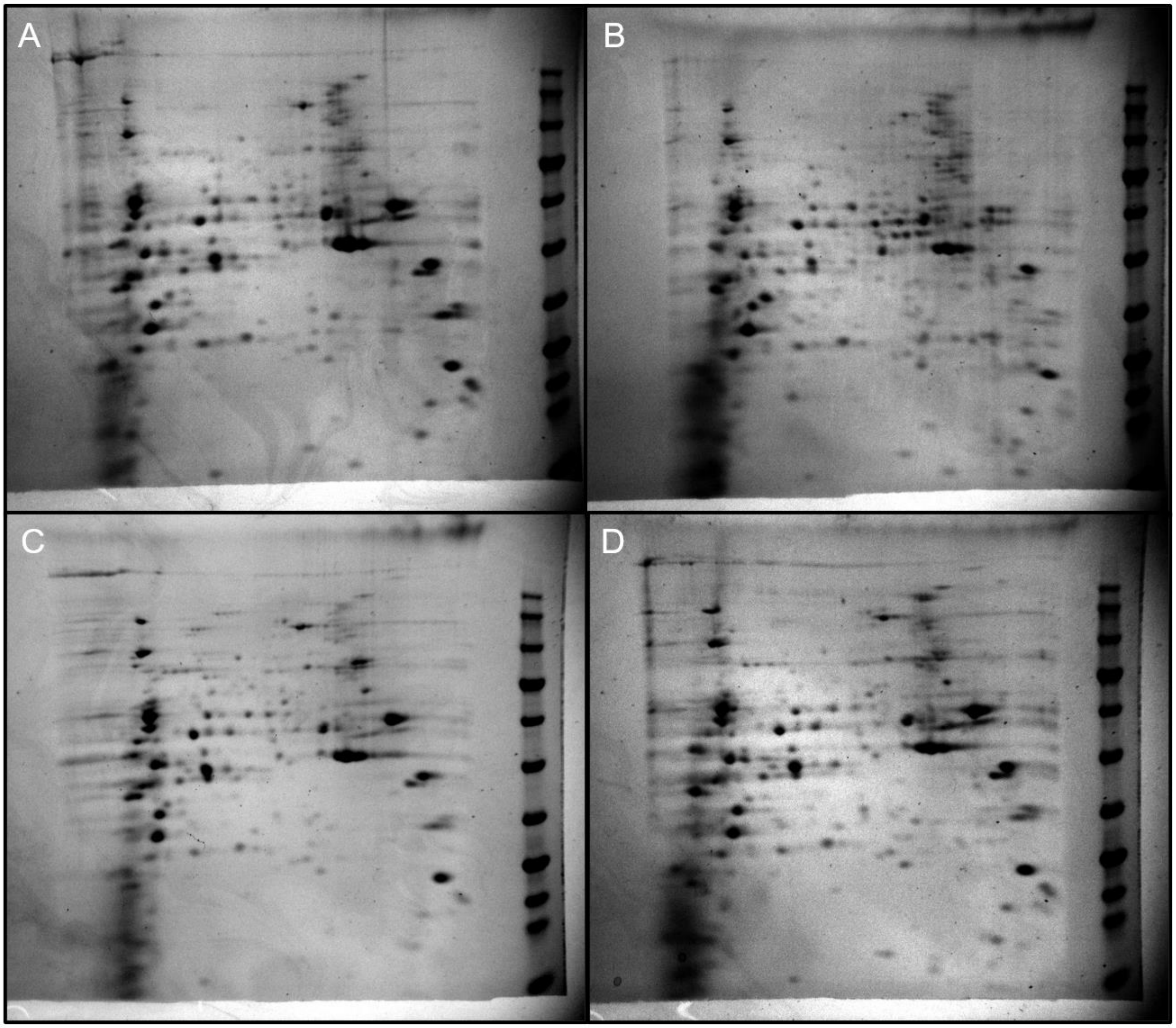


**Supplementary Figure 1.** Representative 2DE gels of (**A**) exercised Pink1, (**B**) non-exercised Pink1, (**C**) exercised wildtype, (**D**) non-exercised wildtype *Drosophila melanogaster.*
