# Supplementary material for: The dysregulated Pink1- Drosophila mitochondrial proteome is partially corrected with exercise
Source: Aging (Albany NY). 2021 Jun 1;13(11):14709–28. doi: 10.18632/aging.203128 (PMC8221352; doi:10.18632/aging.203128)
Supplement: Supplementary Table 1 [file aging-13-203128-s003.docx]

**Supplementary Table 1. Label-free MS identified 516 proteins from the mitochondrial fractions of *Pink1^-^* and WT *D. melanogaster***

| **Identifier** | **Protein name** |
| --- | --- |
| Q94523 | Succinate dehydrogenase [ubiquinone] flavoprotein subunit mitochondrial OS=Drosophila melanogaster OX=7227 GN=SdhA PE=2 SV=3 |
| P29844 | Endoplasmic reticulum chaperone BiP OS=Drosophila melanogaster OX=7227 GN=Hsc70-3 PE=1 SV=2 |
| Q7JUS9 | MIP16332p OS=Drosophila melanogaster OX=7227 GN=Mpcp1 PE=1 SV=1 |
| Q27597 | NADPH--cytochrome P450 reductase OS=Drosophila melanogaster OX=7227 GN=Cpr PE=2 SV=2 |
| Q9W3L4 | GH20802p OS=Drosophila melanogaster OX=7227 GN=Dmel\CG2233 PE=1 SV=1 |
| Q9VIQ8 | Cytochrome c oxidase subunit 4 isoform A OS=Drosophila melanogaster OX=7227 GN=COX4 PE=1 SV=1 |
| Q9VWV6 | Transferrin OS=Drosophila melanogaster OX=7227 GN=Tsf1 PE=1 SV=1 |
| Q9VMS1 | Cyclope isoform A OS=Drosophila melanogaster OX=7227 GN=cype PE=1 SV=3 |
| A0A0B4KHG5 | Chaoptin isoform C OS=Drosophila melanogaster OX=7227 GN=chp PE=1 SV=1 |
| Q9VLJ7 | Fatty acyl-CoA reductase OS=Drosophila melanogaster OX=7227 GN=Sgp PE=2 SV=2 |
| O02649 | Heat shock protein 60A OS=Drosophila melanogaster OX=7227 GN=Hsp60A PE=1 SV=3 |
| A1Z6V5 | FI01422p OS=Drosophila melanogaster OX=7227 GN=Spn43Ab PE=1 SV=1 |
| Q9V4E0 | LD47962p OS=Drosophila melanogaster OX=7227 GN=ND-49 PE=1 SV=2 |
| Q9VMI3 | NADH dehydrogenase [ubiquinone] flavoprotein 1 mitochondrial OS=Drosophila melanogaster OX=7227 GN=ND-51 PE=1 SV=1 |
| Q9W303 | Chitinase-like protein Idgf4 OS=Drosophila melanogaster OX=7227 GN=Idgf4 PE=2 SV=1 |
| Q7JR58 | Enoyl-CoA hydratase short chain 1 isoform A OS=Drosophila melanogaster OX=7227 GN=Echs1 PE=1 SV=1 |
| Q3YMU0 | Protein disulfide-isomerase (Fragment) OS=Drosophila melanogaster OX=7227 GN=ERp60 PE=1 SV=1 |
| Q9VAY2 | Glycoprotein 93 OS=Drosophila melanogaster OX=7227 GN=Gp93 PE=1 SV=1 |
| Q9W402 | NADH dehydrogenase (Ubiquinone) B16.6 subunit isoform A OS=Drosophila melanogaster OX=7227 GN=ND-B16.6 PE=1 SV=1 |
| A0A0B4K715 | Ryanodine receptor isoform J OS=Drosophila melanogaster OX=7227 GN=RyR PE=1 SV=1 |
| Q9VG86 | Fatty acyl-CoA reductase OS=Drosophila melanogaster OX=7227 GN=anon-WO0140519.58 PE=1 SV=3 |
| M9PD14 | Flightin isoform B OS=Drosophila melanogaster OX=7227 GN=fln PE=4 SV=1 |
| Q9VXI6 | Cytochrome b-c1 complex subunit 7 OS=Drosophila melanogaster OX=7227 GN=UQCR-14 PE=1 SV=1 |
| Q9VMN5 | 60 kDa heat shock protein homolog 2 mitochondrial OS=Drosophila melanogaster OX=7227 GN=Hsp60C PE=2 SV=2 |
| Q7KUB1 | Isocitrate dehydrogenase [NADP] OS=Drosophila melanogaster OX=7227 GN=Idh PE=1 SV=1 |
| X2JFD6 | Peroxisomal multifunctional enzyme type 2 isoform B OS=Drosophila melanogaster OX=7227 GN=Mfe2 PE=4 SV=1 |
| Q9VN21 | LD30155p OS=Drosophila melanogaster OX=7227 GN=lost PE=1 SV=1 |
| Q9W3X6 | FI05334p OS=Drosophila melanogaster OX=7227 GN=Fum1 PE=1 SV=3 |
| Q9VQD7 | NADH dehydrogenase [ubiquinone] 1 alpha subcomplex subunit 12 OS=Drosophila melanogaster OX=7227 GN=ND-B17.2 PE=1 SV=1 |
| E2QCS7 | AB hydrolase-1 domain-containing protein OS=Drosophila melanogaster OX=7227 GN=Dmel\CG17097 PE=1 SV=1 |
| Q9VVU1 | FI09602p OS=Drosophila melanogaster OX=7227 GN=GH07925p PE=1 SV=1 |
| Q9VMV9 | Reticulon-like protein OS=Drosophila melanogaster OX=7227 GN=Rtnl1 PE=1 SV=2 |
| Q9VG81 | RH49330p OS=Drosophila melanogaster OX=7227 GN=Sccpdh2 PE=1 SV=1 |
| Q9VZL3 | Sc2 OS=Drosophila melanogaster OX=7227 GN=Sc2 PE=1 SV=1 |
| P00408 | Cytochrome c oxidase subunit 2 OS=Drosophila melanogaster OX=7227 GN=mt:CoII PE=3 SV=1 |
| P36188 | Troponin I OS=Drosophila melanogaster OX=7227 GN=wupA PE=2 SV=3 |
| X2JJG8 | Glutamine synthetase OS=Drosophila melanogaster OX=7227 GN=Gs2 PE=1 SV=1 |
| Q7K511 | CG3835-RA OS=Drosophila melanogaster OX=7227 GN=D2hgdh PE=1 SV=1 |
| Q7KRU8 | Ferritin OS=Drosophila melanogaster OX=7227 GN=Fer1HCH PE=1 SV=1 |
| Q7KN62 | Transitional endoplasmic reticulum ATPase TER94 OS=Drosophila melanogaster OX=7227 GN=TER94 PE=1 SV=1 |
| Q9VN13 | Sideroflexin-1-3 OS=Drosophila melanogaster OX=7227 GN=Sfxn1-3 PE=2 SV=1 |
| Q9W1F7 | IP15825p OS=Drosophila melanogaster OX=7227 GN=Stoml2 PE=1 SV=2 |
| P06002 | Opsin Rh1 OS=Drosophila melanogaster OX=7227 GN=ninaE PE=1 SV=1 |
| Q9W0M4 | LP10861p OS=Drosophila melanogaster OX=7227 GN=Dmel\CG13887 PE=1 SV=1 |
| Q9VS34 | 60S ribosomal protein L18 OS=Drosophila melanogaster OX=7227 GN=RpL18 PE=1 SV=1 |
| Q9VW68 | Gamma-aminobutyric acid transaminase isoform A OS=Drosophila melanogaster OX=7227 GN=Gabat PE=1 SV=1 |
| Q9VWI0 | NADH dehydrogenase (Ubiquinone) 18 kDa subunit OS=Drosophila melanogaster OX=7227 GN=ND-18 PE=1 SV=2 |
| Q9U1L2 | EG:BACR7A4.14 protein OS=Drosophila melanogaster OX=7227 GN=EG:BACR7A4.14 PE=1 SV=1 |
| Q9VQR2 | CG8844 protein OS=Drosophila melanogaster OX=7227 GN=ND-PDSW PE=1 SV=1 |
| Q9VLL3 | A-kinase anchor protein 200 OS=Drosophila melanogaster OX=7227 GN=Akap200 PE=1 SV=3 |
| Q9V9W3 | RE08669p OS=Drosophila melanogaster OX=7227 GN=RpL6 PE=1 SV=1 |
| Q9V4N3 | Cytochrome b5 OS=Drosophila melanogaster OX=7227 GN=Cyt-b5 PE=2 SV=1 |
| Q27606 | Cytochrome P450 4e2 OS=Drosophila melanogaster OX=7227 GN=Cyp4e2 PE=2 SV=2 |
| Q8IQW2 | Cytochrome c oxidase subunit OS=Drosophila melanogaster OX=7227 GN=COX6B PE=1 SV=1 |
| Q9W457 | Serine hydroxymethyltransferase OS=Drosophila melanogaster OX=7227 GN=Shmt PE=1 SV=1 |
| Q9VN86 | AT14148p OS=Drosophila melanogaster OX=7227 GN=Sccpdh1 PE=1 SV=1 |
| P29845 | Heat shock 70 kDa protein cognate 5 OS=Drosophila melanogaster OX=7227 GN=Hsc70-5 PE=1 SV=2 |
| P20432 | Glutathione S-transferase D1 OS=Drosophila melanogaster OX=7227 GN=GstD1 PE=1 SV=1 |
| Q9VY04 | Fezzik isoform A OS=Drosophila melanogaster OX=7227 GN=fiz PE=3 SV=1 |
| M9NEQ9 | Ribosomal protein S10b isoform D OS=Drosophila melanogaster OX=7227 GN=RpS10b PE=1 SV=1 |
| Q9V7Y2 | Sphingosine-1-phosphate lyase OS=Drosophila melanogaster OX=7227 GN=Sply PE=1 SV=1 |
| Q9VEY5 | MICOS complex subunit OS=Drosophila melanogaster OX=7227 GN=Dmel\CG5903 PE=1 SV=1 |
| A0A0B4LHS1 | Myosin alkali light chain 1 isoform D OS=Drosophila melanogaster OX=7227 GN=Mlc1 PE=1 SV=1 |
| E1JJ68 | Rm62 isoform H OS=Drosophila melanogaster OX=7227 GN=Rm62 PE=1 SV=1 |
| C0HKA1 | 40S ribosomal protein S14b OS=Drosophila melanogaster OX=7227 GN=RpS14b PE=2 SV=1 |
| Q9VXM4 | LD12946p OS=Drosophila melanogaster OX=7227 GN=MSBP PE=1 SV=1 |
| Q9W5R8 | 60S ribosomal protein L5 OS=Drosophila melanogaster OX=7227 GN=RpL5 PE=1 SV=2 |
| Q9VG82 | Probable cytochrome P450 9f2 OS=Drosophila melanogaster OX=7227 GN=Cyp9f2 PE=2 SV=1 |
| P29327 | 40S ribosomal protein S6 OS=Drosophila melanogaster OX=7227 GN=RpS6 PE=1 SV=1 |
| Q6IHY5 | HDC00331 OS=Drosophila melanogaster OX=7227 GN=Dmel\CG34172 PE=1 SV=1 |
| Q9VRJ4 | SD02021p OS=Drosophila melanogaster OX=7227 GN=Dmel\CG10672 PE=1 SV=1 |
| Q24046 | Sodium/potassium-transporting ATPase subunit beta-1 OS=Drosophila melanogaster OX=7227 GN=nrv1 PE=1 SV=2 |
| M9PCC1 | Receptor of activated protein kinase C 1 isoform C OS=Drosophila melanogaster OX=7227 GN=Rack1 PE=4 SV=1 |
| Q7YTZ4 | GH22187p OS=Drosophila melanogaster OX=7227 GN=Dmel\CG10737 PE=1 SV=1 |
| Q9V419 | Probable cytochrome P450 28a5 OS=Drosophila melanogaster OX=7227 GN=Cyp28a5 PE=2 SV=1 |
| Q9V438 | Protein disulfide-isomerase A6 homolog OS=Drosophila melanogaster OX=7227 GN=CaBP1 PE=1 SV=1 |
| Q7KW39 | Probable methylmalonate-semialdehyde dehydrogenase [acylating] mitochondrial OS=Drosophila melanogaster OX=7227 GN=CG17896 PE=2 SV=1 |
| Q9VQM2 | NADH dehydrogenase [ubiquinone] 1 subunit C2 OS=Drosophila melanogaster OX=7227 GN=ND-B14.5B PE=1 SV=1 |
| Q9VC18 | RE29555p OS=Drosophila melanogaster OX=7227 GN=BEST:GH11240 PE=1 SV=1 |
| Q9VY05 | GH11762p OS=Drosophila melanogaster OX=7227 GN=Dmel\CG9512 PE=1 SV=1 |
| P21187 | Polyadenylate-binding protein OS=Drosophila melanogaster OX=7227 GN=pAbp PE=1 SV=3 |
| Q9V4I1 | Cytochrome P450 9b2 OS=Drosophila melanogaster OX=7227 GN=Cyp9b2 PE=2 SV=1 |
| A0A0B4LFR4 | Dolichyl-diphosphooligosaccharide--protein glycosyltransferase subunit 2 OS=Drosophila melanogaster OX=7227 GN=OstDelta PE=1 SV=1 |
| Q9VG87 | Fatty acyl-CoA reductase OS=Drosophila melanogaster OX=7227 GN=CG32919 PE=2 SV=2 |
| Q95SI7 | GH23390p OS=Drosophila melanogaster OX=7227 GN=Dmel\CG6028 PE=1 SV=1 |
| Q9W4N8 | LD30122p OS=Drosophila melanogaster OX=7227 GN=Vap33 PE=1 SV=1 |
| Q9VCR9 | RH48101p OS=Drosophila melanogaster OX=7227 GN=Dmel\CG17121 PE=1 SV=1 |
| Q8T3U2 | 40S ribosomal protein S23 OS=Drosophila melanogaster OX=7227 GN=RpS23 PE=1 SV=1 |
| Q86BQ3 | Uncharacterized protein isoform A OS=Drosophila melanogaster OX=7227 GN=Dmel\CG13284 PE=3 SV=1 |
| Q9VZ49 | Poly(U)-specific endoribonuclease homolog OS=Drosophila melanogaster OX=7227 GN=CG2145 PE=2 SV=1 |
| A0A0B4JD95 | Zipper isoform H OS=Drosophila melanogaster OX=7227 GN=zip PE=1 SV=1 |
| Q9VE00 | Probable cytochrome P450 12a4 mitochondrial OS=Drosophila melanogaster OX=7227 GN=Cyp12a4 PE=2 SV=2 |
| E1JHQ1 | Glutamine synthetase OS=Drosophila melanogaster OX=7227 GN=Gs1 PE=1 SV=1 |
| A0A0B4KHJ5 | Glycogen [starch] synthase OS=Drosophila melanogaster OX=7227 GN=GlyS PE=1 SV=1 |
| Q9VU35 | RH34413p OS=Drosophila melanogaster OX=7227 GN=Dmel\CG11267 PE=1 SV=1 |
| Q7KTC7 | Multidrug-Resistance like protein 1 isoform C OS=Drosophila melanogaster OX=7227 GN=MRP PE=1 SV=1 |
| A0A0B4LGZ5 | Ribosomal protein L11 isoform B OS=Drosophila melanogaster OX=7227 GN=RpL11 PE=1 SV=1 |
| Q7KVB1 | ATP binding cassette subfamily B member 7 isoform B OS=Drosophila melanogaster OX=7227 GN=ABCB7 PE=1 SV=1 |
| Q9VCC6 | GM05240p OS=Drosophila melanogaster OX=7227 GN=Dmel\CG6178 PE=1 SV=1 |
| Q9V3W0 | RutC family protein UK114 OS=Drosophila melanogaster OX=7227 GN=UK114 PE=2 SV=1 |
| Q8SXQ1 | Aldehyde dehydrogenase 7 family member A1 OS=Drosophila melanogaster OX=7227 GN=Aldh7A1 PE=1 SV=1 |
| Q9VZS5 | 60S ribosomal protein L28 OS=Drosophila melanogaster OX=7227 GN=RpL28 PE=1 SV=1 |
| M9NFR5 | Ypsilon schachtel isoform B OS=Drosophila melanogaster OX=7227 GN=yps PE=1 SV=1 |
| Q9Y114 | BcDNA.GH10229 OS=Drosophila melanogaster OX=7227 GN=anon-WO0172774.24 PE=1 SV=1 |
| Q8MSU3 | Putative ferric-chelate reductase 1 homolog OS=Drosophila melanogaster OX=7227 GN=CG8399 PE=2 SV=1 |
| Q9W1G0 | Probable transaldolase OS=Drosophila melanogaster OX=7227 GN=Taldo PE=2 SV=2 |
| Q7K3N4 | GH26015p OS=Drosophila melanogaster OX=7227 GN=Dmel\CG8888 PE=1 SV=1 |
| A0A140SRF8 | Uncharacterized protein isoform C OS=Drosophila melanogaster OX=7227 GN=Dmel\CG11857 PE=1 SV=1 |
| E1JJM9 | GEO07866p1 OS=Drosophila melanogaster OX=7227 GN=RpS15Aa PE=1 SV=1 |
| Q7JYW9 | Phosphotransferase OS=Drosophila melanogaster OX=7227 GN=Hex-C PE=1 SV=1 |
| O61491 | Flotillin-1 OS=Drosophila melanogaster OX=7227 GN=Flo1 PE=2 SV=1 |
| E1JJF9 | Neuroglian isoform D OS=Drosophila melanogaster OX=7227 GN=Nrg PE=1 SV=1 |
| P02515 | Heat shock protein 22 OS=Drosophila melanogaster OX=7227 GN=Hsp22 PE=1 SV=4 |
| Q7JXC4 | CG6459 protein OS=Drosophila melanogaster OX=7227 GN=P32 PE=1 SV=1 |
| Q7K485 | CathD isoform A OS=Drosophila melanogaster OX=7227 GN=cathD PE=1 SV=1 |
| X2JC55 | Phosphatidate cytidylyltransferase OS=Drosophila melanogaster OX=7227 GN=Cds PE=1 SV=1 |
| Q9W4P5 | V-type proton ATPase subunit d 1 OS=Drosophila melanogaster OX=7227 GN=VhaAC39-1 PE=2 SV=1 |
| P50882 | 60S ribosomal protein L9 OS=Drosophila melanogaster OX=7227 GN=RpL9 PE=1 SV=2 |
| A4V449 | NADH dehydrogenase (Ubiquinone) 75 kDa subunit isoform B OS=Drosophila melanogaster OX=7227 GN=ND-75 PE=1 SV=1 |
| Q9VHW0 | AT07710p OS=Drosophila melanogaster OX=7227 GN=Dmel\CG7910 PE=2 SV=1 |
| Q9VA83 | Ferritin OS=Drosophila melanogaster OX=7227 GN=Fer2LCH PE=1 SV=1 |
| Q9VLB7 | Rab GDP dissociation inhibitor OS=Drosophila melanogaster OX=7227 GN=Gdi PE=1 SV=1 |
| Q8IMI7 | 40S ribosomal protein S7 OS=Drosophila melanogaster OX=7227 GN=RpS7 PE=1 SV=2 |
| C7LA75 | Heat shock protein cognate 4 isoform G OS=Drosophila melanogaster OX=7227 GN=Hsc70-4 PE=1 SV=1 |
| Q9VL70 | HL08109p OS=Drosophila melanogaster OX=7227 GN=yip2 PE=1 SV=1 |
| O16797 | 60S ribosomal protein L3 OS=Drosophila melanogaster OX=7227 GN=RpL3 PE=1 SV=3 |
| Q9W3N9 | LD24105p OS=Drosophila melanogaster OX=7227 GN=154229_at PE=1 SV=1 |
| A0A0B4K7L1 | ATP-dependent 6-phosphofructokinase OS=Drosophila melanogaster OX=7227 GN=Pfk PE=1 SV=1 |
| P19107 | Phosrestin-1 OS=Drosophila melanogaster OX=7227 GN=Arr2 PE=1 SV=2 |
| Q7PLB8 | Fatty acid synthase 3 OS=Drosophila melanogaster OX=7227 GN=FASN3 PE=1 SV=3 |
| Q9VKT1 | GH11711p OS=Drosophila melanogaster OX=7227 GN=CG17093 PE=1 SV=2 |
| Q9VFP1 | Probable cytochrome P450 6d5 OS=Drosophila melanogaster OX=7227 GN=Cyp6d5 PE=2 SV=1 |
| Q960W6 | Putative fatty acyl-CoA reductase CG8306 OS=Drosophila melanogaster OX=7227 GN=CG8306 PE=2 SV=1 |
| Q9VGF7 | Glutamate carrier 1 isoform A OS=Drosophila melanogaster OX=7227 GN=GC1 PE=1 SV=1 |
| Q9VQL6 | Fatty acid synthase 2 isoform A OS=Drosophila melanogaster OX=7227 GN=FASN2 PE=1 SV=1 |
| Q24319 | Dolichyl-diphosphooligosaccharide--protein glycosyltransferase 48 kDa subunit OS=Drosophila melanogaster OX=7227 GN=Ost48 PE=2 SV=2 |
| Q8SY19 | Microsomal glutathione S-transferase-like isoform A OS=Drosophila melanogaster OX=7227 GN=Mgstl PE=1 SV=1 |
| Q7KV27 | Uncharacterized protein isoform A OS=Drosophila melanogaster OX=7227 GN=ALT PE=1 SV=1 |
| Q9VVW3 | Sideroflexin-2 OS=Drosophila melanogaster OX=7227 GN=Sfxn2 PE=2 SV=2 |
| A0A0B4KH34 | Annexin OS=Drosophila melanogaster OX=7227 GN=AnxB9 PE=1 SV=1 |
| Q9W125 | NADH dehydrogenase [ubiquinone] 1 alpha subcomplex subunit 8 OS=Drosophila melanogaster OX=7227 GN=ND-19 PE=1 SV=1 |
| Q9VSA3 | Probable medium-chain specific acyl-CoA dehydrogenase mitochondrial OS=Drosophila melanogaster OX=7227 GN=CG12262 PE=2 SV=1 |
| P20228 | Glutamate decarboxylase OS=Drosophila melanogaster OX=7227 GN=Gad1 PE=2 SV=2 |
| Q9VZF6 | GH04863p OS=Drosophila melanogaster OX=7227 GN=Dmel\CG14997 PE=1 SV=1 |
| Q9VES7 | Fatty acyl-CoA reductase OS=Drosophila melanogaster OX=7227 GN=Dmel\CG17562 PE=2 SV=2 |
| Q9VMB9 | Cytochrome c oxidase subunit 5B isoform A OS=Drosophila melanogaster OX=7227 GN=COX5B PE=1 SV=1 |
| Q9V771 | Probable cytochrome P450 6a23 OS=Drosophila melanogaster OX=7227 GN=Cyp6a23 PE=2 SV=2 |
| H8F4T6 | FI19911p1 OS=Drosophila melanogaster OX=7227 GN=CG16935-RA PE=1 SV=1 |
| Q23997 | Imaginal disk growth factor 6 OS=Drosophila melanogaster OX=7227 GN=Idgf6 PE=1 SV=2 |
| Q9VAL7 | Calnexin 99A isoform A OS=Drosophila melanogaster OX=7227 GN=Cnx99A PE=1 SV=2 |
| O96553 | C-1-tetrahydrofolate synthase cytoplasmic OS=Drosophila melanogaster OX=7227 GN=pug PE=2 SV=4 |
| Q9VXI1 | Beta hydroxy acid dehydrogenase 1 isoform A OS=Drosophila melanogaster OX=7227 GN=Had1 PE=1 SV=2 |
| Q9VV75 | AT02348p OS=Drosophila melanogaster OX=7227 GN=UQCR-C2 PE=1 SV=1 |
| O46199 | Accessory gland protein Acp53Ea OS=Drosophila melanogaster OX=7227 GN=Acp53Ea PE=2 SV=1 |
| P07486 | Glyceraldehyde-3-phosphate dehydrogenase 1 OS=Drosophila melanogaster OX=7227 GN=Gapdh1 PE=2 SV=2 |
| Q9W3X7 | NADH dehydrogenase [ubiquinone] 1 beta subcomplex subunit 8 mitochondrial OS=Drosophila melanogaster OX=7227 GN=ND-ASHI PE=1 SV=1 |
| Q9W227 | Peptidyl-prolyl cis-trans isomerase OS=Drosophila melanogaster OX=7227 GN=Dmel\CG2852 PE=1 SV=1 |
| Q7K2P3 | GH20817p OS=Drosophila melanogaster OX=7227 GN=Dmel\CG1648 PE=1 SV=1 |
| Q9VKU1 | Fatty acid transport protein 1 isoform A OS=Drosophila melanogaster OX=7227 GN=Fatp1 PE=1 SV=2 |
| Q0E8X7 | Reduction of Rh1 isoform A OS=Drosophila melanogaster OX=7227 GN=roh PE=1 SV=1 |
| Q9VFF0 | GH01077p OS=Drosophila melanogaster OX=7227 GN=UQCR-C1 PE=1 SV=2 |
| Q7KB18 | Epoxide hydrolase OS=Drosophila melanogaster OX=7227 GN=Jheh2 PE=1 SV=1 |
| Q7JV23 | Acetyl-CoA carboxylase isoform B OS=Drosophila melanogaster OX=7227 GN=ACC PE=1 SV=1 |
| Q9VVH5 | AT13736p OS=Drosophila melanogaster OX=7227 GN=UQCR-Q PE=1 SV=1 |
| Q9V8M5 | Probable 3-hydroxyisobutyrate dehydrogenase mitochondrial OS=Drosophila melanogaster OX=7227 GN=CG15093 PE=2 SV=2 |
| Q9I7S8 | Multifunctional protein ADE2 OS=Drosophila melanogaster OX=7227 GN=ade5 PE=2 SV=2 |
| P17336 | Catalase OS=Drosophila melanogaster OX=7227 GN=Cat PE=1 SV=2 |
| Q9VU17 | HL01062p OS=Drosophila melanogaster OX=7227 GN=CT30701 PE=1 SV=1 |
| A0A0B4KHJ9 | Tropomyosin 2 isoform E OS=Drosophila melanogaster OX=7227 GN=Tm2 PE=1 SV=1 |
| Q0E8E8 | MIP08013p1 OS=Drosophila melanogaster OX=7227 GN=Mpcp2 PE=1 SV=1 |
| Q9VJ28 | FI05204p OS=Drosophila melanogaster OX=7227 GN=L2HGDH PE=1 SV=1 |
| X2JGP4 | Protein disulfide-isomerase OS=Drosophila melanogaster OX=7227 GN=Pdi PE=3 SV=1 |
| M9PH10 | Comatose isoform B OS=Drosophila melanogaster OX=7227 GN=comt PE=1 SV=1 |
| P48602 | V-type proton ATPase catalytic subunit A isoform 1 OS=Drosophila melanogaster OX=7227 GN=Vha68-1 PE=2 SV=2 |
| Q8STG9 | DSec61alpha OS=Drosophila melanogaster OX=7227 GN=Sec61alpha PE=1 SV=1 |
| Q7KTJ7 | Basigin isoform G OS=Drosophila melanogaster OX=7227 GN=Bsg PE=1 SV=1 |
| P50887 | 60S ribosomal protein L22 OS=Drosophila melanogaster OX=7227 GN=RpL22 PE=1 SV=2 |
| A0A0B4LGS4 | Vacuolar H[+]-ATPase 26kD subunit isoform C OS=Drosophila melanogaster OX=7227 GN=Vha26 PE=3 SV=1 |
| P07190 | Maltase A1 OS=Drosophila melanogaster OX=7227 GN=Mal-A1 PE=2 SV=2 |
| Q9VGQ4 | GH25284p OS=Drosophila melanogaster OX=7227 GN=scpr-B PE=2 SV=2 |
| Q8MKK5 | GH09271p OS=Drosophila melanogaster OX=7227 GN=kcc PE=1 SV=1 |
| Q9VKM3 | ATP synthase subunit OS=Drosophila melanogaster OX=7227 GN=ATPsynG PE=1 SV=1 |
| Q0E9B6 | 40S ribosomal protein S11 OS=Drosophila melanogaster OX=7227 GN=RpS11 PE=1 SV=1 |
| Q9VE75 | V-type proton ATPase subunit a OS=Drosophila melanogaster OX=7227 GN=Vha100-2 PE=1 SV=2 |
| D4G7B1 | Imaginal disc growth factor 2 isoform B OS=Drosophila melanogaster OX=7227 GN=Idgf2 PE=1 SV=1 |
| P91929 | NADH dehydrogenase [ubiquinone] 1 alpha subcomplex subunit 10 mitochondrial OS=Drosophila melanogaster OX=7227 GN=ND-42 PE=2 SV=2 |
| P46223 | 60S ribosomal protein L7a OS=Drosophila melanogaster OX=7227 GN=RpL7A PE=1 SV=2 |
| Q7K188 | Protein quiver OS=Drosophila melanogaster OX=7227 GN=Dmel\CG6329 PE=2 SV=1 |
| A0A0B4KFE4 | Acyl-CoA synthetase long-chain isoform J OS=Drosophila melanogaster OX=7227 GN=Acsl PE=1 SV=1 |
| Q9V3A8 | Ergic53 isoform A OS=Drosophila melanogaster OX=7227 GN=ergic53 PE=1 SV=1 |
| Q9V3P0 | Peroxiredoxin 1 OS=Drosophila melanogaster OX=7227 GN=Jafrac1 PE=1 SV=1 |
| E1JJA4 | Shibire isoform L OS=Drosophila melanogaster OX=7227 GN=shi PE=1 SV=1 |
| Q94514 | Cytochrome c oxidase subunit 5A mitochondrial OS=Drosophila melanogaster OX=7227 GN=COX5A PE=2 SV=2 |
| C9QP21 | Carboxylic ester hydrolase OS=Drosophila melanogaster OX=7227 GN=Est-6 PE=1 SV=1 |
| A8DYI6 | Prohibitin 2 isoform E OS=Drosophila melanogaster OX=7227 GN=Phb2 PE=1 SV=1 |
| P21914 | Succinate dehydrogenase [ubiquinone] iron-sulfur subunit mitochondrial OS=Drosophila melanogaster OX=7227 GN=SdhB PE=2 SV=2 |
| A8Y535 | Cytochrome b-c1 complex subunit 6 OS=Drosophila melanogaster OX=7227 GN=UQCR-11 PE=1 SV=2 |
| Q9VQ61 | Aspartate aminotransferase OS=Drosophila melanogaster OX=7227 GN=Got2 PE=1 SV=1 |
| P16378 | G protein alpha o subunit OS=Drosophila melanogaster OX=7227 GN=Galphao PE=1 SV=1 |
| Q9VNX4 | Multifunctional fusion protein OS=Drosophila melanogaster OX=7227 GN=P5CDh1 PE=1 SV=1 |
| A1Z8N1 | Facilitated trehalose transporter Tret1-1 OS=Drosophila melanogaster OX=7227 GN=Tret1-1 PE=1 SV=1 |
| Q7KLX3 | GEO05407p1 OS=Drosophila melanogaster OX=7227 GN=Tapdelta PE=1 SV=1 |
| Q9VAJ9 | SD09259p OS=Drosophila melanogaster OX=7227 GN=Dmel\CG1907 PE=1 SV=1 |
| Q9W3F3 | RH03540p OS=Drosophila melanogaster OX=7227 GN=Dmel\CG15347 PE=1 SV=2 |
| Q76NQ0 | Dolichyl-diphosphooligosaccharide--protein glycosyltransferase subunit 1 OS=Drosophila melanogaster OX=7227 GN=CG5364 PE=1 SV=1 |
| O76742 | CG5915 protein OS=Drosophila melanogaster OX=7227 GN=Rab7 PE=1 SV=1 |
| B7YZQ3 | Prominin isoform D OS=Drosophila melanogaster OX=7227 GN=prom PE=1 SV=1 |
| E2QD65 | Ribosomal protein S19a isoform C OS=Drosophila melanogaster OX=7227 GN=RpS19a PE=1 SV=1 |
| B7Z0L1 | Fasciclin 1 isoform E OS=Drosophila melanogaster OX=7227 GN=Fas1 PE=1 SV=1 |
| Q9Y112 | BcDNA.GH10614 OS=Drosophila melanogaster OX=7227 GN=dm1 PE=1 SV=1 |
| Q500Y7 | GEO11443p1 OS=Drosophila melanogaster OX=7227 GN=UQCR-6.4 PE=2 SV=1 |
| P41073 | Zinc finger protein on ecdysone puffs OS=Drosophila melanogaster OX=7227 GN=Pep PE=1 SV=1 |
| E1JI91 | UTP--glucose-1-phosphate uridylyltransferase OS=Drosophila melanogaster OX=7227 GN=UGP PE=1 SV=1 |
| P09180 | 60S ribosomal protein L4 OS=Drosophila melanogaster OX=7227 GN=RpL4 PE=1 SV=2 |
| Q7KSQ0 | LD46175p OS=Drosophila melanogaster OX=7227 GN=sea PE=1 SV=1 |
| Q9W2H8 | Uncharacterized protein isoform B OS=Drosophila melanogaster OX=7227 GN=BEST:GH22856 PE=1 SV=3 |
| C0PDF4 | MIP06482p OS=Drosophila melanogaster OX=7227 GN=CG18815-RA PE=1 SV=1 |
| Q9VIB5 | Carboxylic ester hydrolase OS=Drosophila melanogaster OX=7227 GN=alpha-Est7 PE=1 SV=1 |
| Q9VPR1 | GH02075p OS=Drosophila melanogaster OX=7227 GN=Tspo PE=1 SV=1 |
| P38979 | 40S ribosomal protein SA OS=Drosophila melanogaster OX=7227 GN=sta PE=1 SV=3 |
| C0HK92 | Uncharacterized protein CG45076 OS=Drosophila melanogaster OX=7227 GN=CG45076 PE=1 SV=1 |
| E2QCF1 | ATP-citrate synthase OS=Drosophila melanogaster OX=7227 GN=ATPCL PE=1 SV=1 |
| Q9W141 | Putative ATP synthase subunit f mitochondrial OS=Drosophila melanogaster OX=7227 GN=CG4692 PE=3 SV=1 |
| Q9V3S9 | Very long-chain-fatty-acid--CoA ligase bubblegum OS=Drosophila melanogaster OX=7227 GN=bgm PE=1 SV=1 |
| M9PGL7 | Translocase of outer membrane 40 isoform C OS=Drosophila melanogaster OX=7227 GN=Tom40 PE=4 SV=1 |
| A0A0B4LFL2 | Eukaryotic translation initiation factor 3 subunit C OS=Drosophila melanogaster OX=7227 GN=eIF3c PE=3 SV=1 |
| A0A0B4KEL0 | Aldehyde dehydrogenase OS=Drosophila melanogaster OX=7227 GN=Aldh-III PE=1 SV=1 |
| M9PJP0 | Nipsnap isoform F OS=Drosophila melanogaster OX=7227 GN=Nipsnap PE=4 SV=2 |
| M9PDK5 | V-type proton ATPase subunit H OS=Drosophila melanogaster OX=7227 GN=VhaSFD PE=3 SV=1 |
| M9PG76 | 60S acidic ribosomal protein P0 OS=Drosophila melanogaster OX=7227 GN=RpLP0 PE=3 SV=1 |
| P08736 | Elongation factor 1-alpha 1 OS=Drosophila melanogaster OX=7227 GN=eEF1alpha1 PE=1 SV=2 |
| Q9VD29 | RE74312p OS=Drosophila melanogaster OX=7227 GN=Sar1 PE=1 SV=1 |
| Q9W1B9 | GEO07602p1 OS=Drosophila melanogaster OX=7227 GN=RpL12 PE=1 SV=1 |
| A0A0B4LG52 | GEO08239p1 OS=Drosophila melanogaster OX=7227 GN=RpS16 PE=2 SV=1 |
| C8VV14 | Fructose-bisphosphate aldolase OS=Drosophila melanogaster OX=7227 GN=Ald1 PE=1 SV=1 |
| Q0KHZ6 | Uncharacterized protein isoform A OS=Drosophila melanogaster OX=7227 GN=ETFB PE=1 SV=1 |
| Q8IN25 | Aminopeptidase OS=Drosophila melanogaster OX=7227 GN=Dmel\CG31198 PE=1 SV=1 |
| P55841 | 60S ribosomal protein L14 OS=Drosophila melanogaster OX=7227 GN=RpL14 PE=1 SV=1 |
| Q06559 | 40S ribosomal protein S3 OS=Drosophila melanogaster OX=7227 GN=RpS3 PE=1 SV=1 |
| M9MSJ3 | Uncharacterized protein isoform B OS=Drosophila melanogaster OX=7227 GN=HDC03337 PE=4 SV=1 |
| Q7K569 | Glycerol-3-phosphate dehydrogenase OS=Drosophila melanogaster OX=7227 GN=Gpo1 PE=1 SV=1 |
| Q9W2E8 | NADH dehydrogenase (Ubiquinone) B12 subunit isoform A OS=Drosophila melanogaster OX=7227 GN=ND-B12 PE=2 SV=1 |
| P13008 | 40S ribosomal protein S26 OS=Drosophila melanogaster OX=7227 GN=RpS26 PE=1 SV=1 |
| Q9VA18 | Coiled-coil-helix-coiled-coil-helix domain containing 3 OS=Drosophila melanogaster OX=7227 GN=Chchd3 PE=1 SV=1 |
| Q9W095 | FI03690p OS=Drosophila melanogaster OX=7227 GN=Gk2 PE=2 SV=2 |
| Q7JYH3 | NADH dehydrogenase (Ubiquinone) B14.7 subunit OS=Drosophila melanogaster OX=7227 GN=ND-B14.7 PE=1 SV=1 |
| Q9W1H8 | GH13256p OS=Drosophila melanogaster OX=7227 GN=Mtpbeta PE=1 SV=1 |
| Q9VGT8 | UDP-glucuronosyltransferase OS=Drosophila melanogaster OX=7227 GN=Ugt35C1 PE=3 SV=2 |
| Q9VTU2 | NADH dehydrogenase (Ubiquinone) SGDH subunit isoform A OS=Drosophila melanogaster OX=7227 GN=ND-SGDH PE=1 SV=1 |
| Q9VM14 | AT21758p OS=Drosophila melanogaster OX=7227 GN=muc PE=1 SV=1 |
| A0A0C4DHG5 | B52 isoform O OS=Drosophila melanogaster OX=7227 GN=B52 PE=4 SV=1 |
| P35381 | ATP synthase subunit alpha mitochondrial OS=Drosophila melanogaster OX=7227 GN=blw PE=1 SV=2 |
| P35415 | Paramyosin long form OS=Drosophila melanogaster OX=7227 GN=Prm PE=1 SV=1 |
| P29613 | Triosephosphate isomerase OS=Drosophila melanogaster OX=7227 GN=Tpi PE=1 SV=3 |
| M9PBL3 | Heat shock protein 83 isoform B OS=Drosophila melanogaster OX=7227 GN=Hsp83 PE=3 SV=1 |
| Q6GUR9 | ACP53C14B OS=Drosophila melanogaster OX=7227 GN=Acp53C14b PE=1 SV=1 |
| P55830 | 40S ribosomal protein S3a OS=Drosophila melanogaster OX=7227 GN=RpS3A PE=1 SV=4 |
| Q9VEX6 | ATPase family AAA domain-containing protein 3A homolog OS=Drosophila melanogaster OX=7227 GN=bor PE=1 SV=2 |
| Q9VHS2 | Cytochrome c oxidase subunit 7A mitochondrial OS=Drosophila melanogaster OX=7227 GN=COX7A PE=3 SV=1 |
| Q9VMT5 | Probable cytochrome P450 28d1 OS=Drosophila melanogaster OX=7227 GN=Cyp28d1 PE=2 SV=1 |
| X2J8Y6 | Accessory gland protein 36DE isoform B OS=Drosophila melanogaster OX=7227 GN=Acp36DE PE=4 SV=1 |
| Q9VXK7 | LD31474p OS=Drosophila melanogaster OX=7227 GN=ND-20 PE=2 SV=1 |
| L0MPS3 | Apolipophorin isoform B OS=Drosophila melanogaster OX=7227 GN=apolpp PE=1 SV=1 |
| Q9VQ29 | Cytochrome b-c1 complex subunit Rieske mitochondrial OS=Drosophila melanogaster OX=7227 GN=RFeSP PE=1 SV=3 |
| B7YZI0 | Vacuolar H[+] ATPase 44kD subunit isoform F OS=Drosophila melanogaster OX=7227 GN=Vha44 PE=1 SV=1 |
| Q9VTB4 | NADH dehydrogenase (Ubiquinone) 13 kDa B subunit OS=Drosophila melanogaster OX=7227 GN=ND-13B PE=1 SV=2 |
| P24156 | Protein l(2)37Cc OS=Drosophila melanogaster OX=7227 GN=l(2)37Cc PE=2 SV=2 |
| Q9VBP6 | Succinate-semialdehyde dehydrogenase OS=Drosophila melanogaster OX=7227 GN=Ssadh PE=1 SV=1 |
| Q9VKI8 | GH03305p OS=Drosophila melanogaster OX=7227 GN=GH26 PE=1 SV=1 |
| Q9VII1 | FI02856p OS=Drosophila melanogaster OX=7227 GN=Dmel\CG9336 PE=1 SV=1 |
| Q9VZU4 | LD25561p OS=Drosophila melanogaster OX=7227 GN=ND-30 PE=1 SV=1 |
| Q9Y166 | BcDNA.GH02431 OS=Drosophila melanogaster OX=7227 GN=Dic1 PE=1 SV=1 |
| O18404 | 3-hydroxyacyl-CoA dehydrogenase type-2 OS=Drosophila melanogaster OX=7227 GN=scu PE=1 SV=1 |
| B6IDY5 | CYP6G1 OS=Drosophila melanogaster OX=7227 GN=Cyp6g1 PE=2 SV=1 |
| Q8I0D4 | RE20510p OS=Drosophila melanogaster OX=7227 GN=Dmel\CG9297 PE=1 SV=1 |
| Q7JQH9 | LD31742p OS=Drosophila melanogaster OX=7227 GN=whd PE=1 SV=1 |
| Q24186 | 40S ribosomal protein S5a OS=Drosophila melanogaster OX=7227 GN=RpS5a PE=1 SV=1 |
| Q24251 | ATP synthase subunit d mitochondrial OS=Drosophila melanogaster OX=7227 GN=ATPsynD PE=2 SV=2 |
| O77460 | Inorganic pyrophosphatase OS=Drosophila melanogaster OX=7227 GN=Nurf-38 PE=1 SV=3 |
| Q9W3K9 | FI02989p OS=Drosophila melanogaster OX=7227 GN=Ldsdh1 PE=1 SV=1 |
| A0A0B4K7C1 | Aspartyl beta-hydroxylase isoform H OS=Drosophila melanogaster OX=7227 GN=Asph PE=1 SV=1 |
| P10676 | Neither inactivation nor afterpotential protein C OS=Drosophila melanogaster OX=7227 GN=ninaC PE=1 SV=2 |
| Q9VM18 | Trehalose 6-phosphate phosphatase OS=Drosophila melanogaster OX=7227 GN=21430192 PE=1 SV=1 |
| P52029 | Glucose-6-phosphate isomerase OS=Drosophila melanogaster OX=7227 GN=Pgi PE=2 SV=2 |
| A4V4F2 | Flotillin 2 isoform F OS=Drosophila melanogaster OX=7227 GN=Flo2 PE=1 SV=1 |
| Q7K8X7 | Glutathione S transferase E9 OS=Drosophila melanogaster OX=7227 GN=GstE9 PE=1 SV=1 |
| Q9VJZ4 | AT12494p OS=Drosophila melanogaster OX=7227 GN=ND-B22 PE=1 SV=1 |
| C6SUW3 | LD13662p OS=Drosophila melanogaster OX=7227 GN=RpS9 PE=1 SV=1 |
| Q9VVH3 | MICOS complex subunit MIC13 homolog QIL1 OS=Drosophila melanogaster OX=7227 GN=QIL1 PE=2 SV=1 |
| Q0E9N2 | Lethal (2) 01289 isoform K OS=Drosophila melanogaster OX=7227 GN=l(2)01289 PE=1 SV=3 |
| Q2QBM1 | Malic enzyme OS=Drosophila melanogaster OX=7227 GN=Men PE=1 SV=1 |
| Q9W2J4 | UDP-glucuronosyltransferase OS=Drosophila melanogaster OX=7227 GN=Ugt49B1 PE=1 SV=2 |
| Q7KE33 | Odorant binding protein c OS=Drosophila melanogaster OX=7227 GN=Obp51a PE=2 SV=1 |
| Q9W2M4 | Uncharacterized protein OS=Drosophila melanogaster OX=7227 GN=Dmel\CG10527 PE=1 SV=1 |
| P54385 | Glutamate dehydrogenase mitochondrial OS=Drosophila melanogaster OX=7227 GN=Gdh PE=1 SV=2 |
| Q9VUC1 | Hsc70Cb isoform A OS=Drosophila melanogaster OX=7227 GN=Hsc70Cb PE=1 SV=1 |
| A0A0B4KEX7 | Uncharacterized protein OS=Drosophila melanogaster OX=7227 GN=Dmel\CG43788 PE=4 SV=1 |
| A0A0B4K6N1 | 40S ribosomal protein S8 OS=Drosophila melanogaster OX=7227 GN=RpS8 PE=3 SV=1 |
| Q9VRL0 | Cytochrome c1 isoform A OS=Drosophila melanogaster OX=7227 GN=Cyt-c1 PE=1 SV=1 |
| Q9VX36 | NADH dehydrogenase (Ubiquinone) 24 kDa subunit isoform A OS=Drosophila melanogaster OX=7227 GN=ND-24 PE=1 SV=1 |
| Q9VB10 | GH01709p OS=Drosophila melanogaster OX=7227 GN=Dmel\CG5590 PE=1 SV=1 |
| Q9VXG9 | GH25683p OS=Drosophila melanogaster OX=7227 GN=Dmel\CG4239 PE=2 SV=1 |
| M9PI17 | Uncharacterized protein isoform D OS=Drosophila melanogaster OX=7227 GN=Dmel\CG6084 PE=1 SV=1 |
| Q7KN94 | Walrus isoform A OS=Drosophila melanogaster OX=7227 GN=wal PE=1 SV=1 |
| X2JGM9 | 40S ribosomal protein S4 OS=Drosophila melanogaster OX=7227 GN=RpS4 PE=3 SV=1 |
| Q7JS69 | FI04632p OS=Drosophila melanogaster OX=7227 GN=nrv3 PE=1 SV=1 |
| P02574 | Actin larval muscle OS=Drosophila melanogaster OX=7227 GN=Act79B PE=1 SV=2 |
| Q9W1X9 | OCIA domain-containing protein 1 OS=Drosophila melanogaster OX=7227 GN=asrij PE=1 SV=1 |
| P29829 | Guanine nucleotide-binding protein subunit beta-2 OS=Drosophila melanogaster OX=7227 GN=Gbeta76C PE=1 SV=3 |
| P31009 | 40S ribosomal protein S2 OS=Drosophila melanogaster OX=7227 GN=RpS2 PE=1 SV=2 |
| Q9XY35 | Cytochrome b-c1 complex subunit 9 OS=Drosophila melanogaster OX=7227 GN=ox PE=3 SV=1 |
| Q9VAC1 | GM14349p OS=Drosophila melanogaster OX=7227 GN=anon-WO0153538.36 PE=1 SV=1 |
| O18335 | Drab11 OS=Drosophila melanogaster OX=7227 GN=Rab11 PE=1 SV=1 |
| Q9VGS3 | RH44771p OS=Drosophila melanogaster OX=7227 GN=SdhC PE=1 SV=2 |
| P83967 | Actin indirect flight muscle OS=Drosophila melanogaster OX=7227 GN=Act88F PE=1 SV=1 |
| Q9VB69 | Malic enzyme OS=Drosophila melanogaster OX=7227 GN=Men-b PE=1 SV=1 |
| Q24439 | ATP synthase subunit O mitochondrial OS=Drosophila melanogaster OX=7227 GN=ATPsynO PE=2 SV=2 |
| P26308 | Guanine nucleotide-binding protein subunit beta-1 OS=Drosophila melanogaster OX=7227 GN=Gbeta13F PE=1 SV=1 |
| A4UZZ4 | Alpha-1 4 glucan phosphorylase OS=Drosophila melanogaster OX=7227 GN=GlyP PE=1 SV=1 |
| Q9VKX2 | Malate dehydrogenase OS=Drosophila melanogaster OX=7227 GN=Mdh1 PE=1 SV=2 |
| Q7K3D4 | Peptidylprolyl isomerase OS=Drosophila melanogaster OX=7227 GN=zda PE=1 SV=1 |
| Q9Y119 | BcDNA.GH08860 OS=Drosophila melanogaster OX=7227 GN=Tps1 PE=1 SV=1 |
| P41093 | 60S ribosomal protein L18a OS=Drosophila melanogaster OX=7227 GN=RpL18A PE=1 SV=1 |
| Q9V397 | BcDNA.GH12558 OS=Drosophila melanogaster OX=7227 GN=Mtpalpha PE=1 SV=1 |
| Q9VZW7 | Carnitine palmitoyltransferase 2 OS=Drosophila melanogaster OX=7227 GN=CPT2 PE=1 SV=1 |
| Q8IRD3 | Glutathione peroxidase OS=Drosophila melanogaster OX=7227 GN=PHGPx PE=1 SV=1 |
| A0A0B4KHD3 | Mitochondrial pyruvate carrier OS=Drosophila melanogaster OX=7227 GN=Mpc1 PE=2 SV=1 |
| Q9VPE2 | NADH dehydrogenase (Ubiquinone) 39 kDa subunit isoform A OS=Drosophila melanogaster OX=7227 GN=ND-39 PE=1 SV=1 |
| Q09332 | UDP-glucose:glycoprotein glucosyltransferase OS=Drosophila melanogaster OX=7227 GN=Ugt PE=1 SV=2 |
| Q9VJ19 | RE25263p OS=Drosophila melanogaster OX=7227 GN=RpL30 PE=1 SV=1 |
| P92177 | 14-3-3 protein epsilon OS=Drosophila melanogaster OX=7227 GN=14-3-3epsilon PE=1 SV=2 |
| P13060 | Elongation factor 2 OS=Drosophila melanogaster OX=7227 GN=EF2 PE=1 SV=4 |
| Q94516 | ATP synthase subunit b mitochondrial OS=Drosophila melanogaster OX=7227 GN=ATPsynB PE=2 SV=2 |
| Q9VUZ0 | Translocon-associated protein subunit beta OS=Drosophila melanogaster OX=7227 GN=SsRbeta PE=1 SV=2 |
| Q6GUT7 | ACP53C14A OS=Drosophila melanogaster OX=7227 GN=Acp53C14a PE=2 SV=1 |
| A1Z9E3 | Elongation factor Tu OS=Drosophila melanogaster OX=7227 GN=mEFTu1 PE=1 SV=1 |
| Q8T4G5 | SD01613p OS=Drosophila melanogaster OX=7227 GN=AFG3L2 PE=1 SV=1 |
| Q02645 | Protein hu-li tai shao OS=Drosophila melanogaster OX=7227 GN=hts PE=1 SV=2 |
| X2JAJ8 | Serpin 38F isoform B OS=Drosophila melanogaster OX=7227 GN=Spn38F PE=3 SV=1 |
| Q9VBR6 | RE74917p OS=Drosophila melanogaster OX=7227 GN=tobi PE=2 SV=1 |
| P41572 | 6-phosphogluconate dehydrogenase decarboxylating OS=Drosophila melanogaster OX=7227 GN=Pgd PE=2 SV=1 |
| P07251 | ATP synthase subunit alpha mitochondrial OS=Saccharomyces cerevisiae (strain ATCC 204508 / S288c) OX=559292 GN=ATP1 PE=1 SV=5 |
| Q7JZK1 | NADH dehydrogenase (Ubiquinone) B14 subunit isoform A OS=Drosophila melanogaster OX=7227 GN=ND-B14 PE=1 SV=1 |
| A1Z7S3 | Rab32 isoform B OS=Drosophila melanogaster OX=7227 GN=Rab32 PE=1 SV=1 |
| Q9V3S0 | Cytochrome P450 4g1 OS=Drosophila melanogaster OX=7227 GN=Cyp4g1 PE=2 SV=1 |
| P22465 | Annexin B10 OS=Drosophila melanogaster OX=7227 GN=AnxB10 PE=2 SV=3 |
| A4V0N4 | MIP16230p OS=Drosophila melanogaster OX=7227 GN=Vha68-2 PE=1 SV=1 |
| P84051 | Histone H2A OS=Drosophila melanogaster OX=7227 GN=His2A PE=1 SV=2 |
| O46067 | EG:25E8.1 protein OS=Drosophila melanogaster OX=7227 GN=GRP170 PE=1 SV=1 |
| Q9VWP2 | RH57257p OS=Drosophila melanogaster OX=7227 GN=Dmel\CG7322 PE=1 SV=1 |
| P41126 | 60S ribosomal protein L13 OS=Drosophila melanogaster OX=7227 GN=RpL13 PE=1 SV=1 |
| Q9V9T5 | GM14617p OS=Drosophila melanogaster OX=7227 GN=Mccc1 PE=1 SV=2 |
| Q9W1N3 | Levy isoform A OS=Drosophila melanogaster OX=7227 GN=levy PE=1 SV=1 |
| O18332 | FI01544p OS=Drosophila melanogaster OX=7227 GN=Rab1 PE=1 SV=1 |
| M9PBV2 | NTPase isoform F OS=Drosophila melanogaster OX=7227 GN=NTPase PE=1 SV=1 |
| Q9VWH4 | Probable isocitrate dehydrogenase [NAD] subunit alpha mitochondrial OS=Drosophila melanogaster OX=7227 GN=l(1)G0156 PE=2 SV=1 |
| A0A0B4KGU9 | Extended synaptotagmin-like protein 2 isoform D OS=Drosophila melanogaster OX=7227 GN=Esyt2 PE=1 SV=1 |
| P84040 | Histone H4 OS=Drosophila melanogaster OX=7227 GN=His4 PE=1 SV=2 |
| A4V0B5 | Nervana 2 isoform F OS=Drosophila melanogaster OX=7227 GN=nrv2 PE=1 SV=1 |
| A0A0B4LHE7 | V-type proton ATPase subunit G OS=Drosophila melanogaster OX=7227 GN=Vha13 PE=3 SV=1 |
| Q9VV42 | HL08057p OS=Drosophila melanogaster OX=7227 GN=Pdh PE=1 SV=1 |
| Q9VY92 | GEO07753p1 OS=Drosophila melanogaster OX=7227 GN=Dmel\CG11151 PE=1 SV=1 |
| Q9VQG4 | Congested-like trachea protein OS=Drosophila melanogaster OX=7227 GN=colt PE=2 SV=1 |
| Q9W229 | 40S ribosomal protein S24 OS=Drosophila melanogaster OX=7227 GN=RpS24 PE=1 SV=1 |
| Q9V9S8 | Ferrochelatase mitochondrial OS=Drosophila melanogaster OX=7227 GN=FeCh PE=2 SV=1 |
| Q9U6L5 | Ejaculatory bulb-specific protein 1 OS=Drosophila melanogaster OX=7227 GN=Ebp PE=1 SV=1 |
| Q9W2L6 | RH02475p OS=Drosophila melanogaster OX=7227 GN=Dmel\CG9394 PE=1 SV=1 |
| Q9W401 | Probable citrate synthase mitochondrial OS=Drosophila melanogaster OX=7227 GN=kdn PE=2 SV=1 |
| Q9VB46 | FI18644p1 OS=Drosophila melanogaster OX=7227 GN=Hmu PE=1 SV=1 |
| P29310 | 14-3-3 protein zeta OS=Drosophila melanogaster OX=7227 GN=14-3-3zeta PE=1 SV=1 |
| Q9VEJ3 | Pyrroline-5-carboxylate reductase OS=Drosophila melanogaster OX=7227 GN=P5cr-2 PE=1 SV=1 |
| Q9VLC5 | Aldehyde dehydrogenase OS=Drosophila melanogaster OX=7227 GN=Aldh PE=1 SV=1 |
| P84029 | Cytochrome c-2 OS=Drosophila melanogaster OX=7227 GN=Cyt-c-p PE=1 SV=2 |
| Q9VGZ3 | Iron regulatory protein 1B OS=Drosophila melanogaster OX=7227 GN=Irp-1B PE=1 SV=1 |
| E1JIJ5 | Vacuolar proton pump subunit B OS=Drosophila melanogaster OX=7227 GN=Vha55 PE=1 SV=1 |
| A8JRB8 | MIP04243p OS=Drosophila melanogaster OX=7227 GN=Dmel\CG5028 PE=1 SV=1 |
| O01666 | ATP synthase subunit gamma mitochondrial OS=Drosophila melanogaster OX=7227 GN=ATPsyngamma PE=2 SV=2 |
| A4UZR3 | Trehalase OS=Drosophila melanogaster OX=7227 GN=Treh PE=1 SV=1 |
| Q76NR6 | Regucalcin isoform D OS=Drosophila melanogaster OX=7227 GN=regucalcin PE=1 SV=1 |
| Q6IDF5 | NADH dehydrogenase (Ubiquinone) B15 subunit OS=Drosophila melanogaster OX=7227 GN=ND-B15 PE=1 SV=1 |
| Q9W3N1 | RH59310p OS=Drosophila melanogaster OX=7227 GN=spidey PE=1 SV=1 |
| Q8MSI2 | GH15296p OS=Drosophila melanogaster OX=7227 GN=Scp1 PE=1 SV=1 |
| P41094 | 40S ribosomal protein S18 OS=Drosophila melanogaster OX=7227 GN=RpS18 PE=1 SV=1 |
| Q7JWF1 | Electron transfer flavoprotein-ubiquinone oxidoreductase isoform A OS=Drosophila melanogaster OX=7227 GN=Etf-QO PE=1 SV=1 |
| Q8SWX4 | Aminopeptidase OS=Drosophila melanogaster OX=7227 GN=CG5839 PE=1 SV=1 |
| Q9VHN7 | LP07963p OS=Drosophila melanogaster OX=7227 GN=anon-WO0118547.344 PE=1 SV=3 |
| M9MRC9 | GEO07462p1 OS=Drosophila melanogaster OX=7227 GN=RpL27A PE=2 SV=1 |
| Q95U15 | GH14252p OS=Drosophila melanogaster OX=7227 GN=Mf PE=1 SV=1 |
| Q7K332 | GH17623p OS=Drosophila melanogaster OX=7227 GN=CG3364 PE=2 SV=1 |
| X2JEA2 | Uncharacterized protein isoform B OS=Drosophila melanogaster OX=7227 GN=Dmel\CG4666 PE=4 SV=1 |
| X2JB48 | Stress-sensitive B isoform E OS=Drosophila melanogaster OX=7227 GN=sesB PE=1 SV=1 |
| Q9VKR4 | Aminomethyltransferase OS=Drosophila melanogaster OX=7227 GN=Dmel\CG6415 PE=1 SV=1 |
| M9MS06 | Alpha actinin isoform D OS=Drosophila melanogaster OX=7227 GN=Actn PE=1 SV=1 |
| Q9VGQ1 | GM01350p OS=Drosophila melanogaster OX=7227 GN=alpha-KGDHC PE=1 SV=1 |
| Q8IPP8 | Uncharacterized protein OS=Drosophila melanogaster OX=7227 GN=Dmel\CG31548 PE=1 SV=1 |
| Q8IQQ0 | Neural conserved at 73EF isoform F OS=Drosophila melanogaster OX=7227 GN=Nc73EF PE=1 SV=1 |
| A1ZA73 | Stretchin-Mlck isoform R OS=Drosophila melanogaster OX=7227 GN=Strn-Mlck PE=1 SV=2 |
| B4ZJ91 | Seminal fluid protein 24Bb OS=Drosophila melanogaster OX=7227 GN=Sfp24Bb PE=2 SV=1 |
| A0A0B4LFB8 | Optic atrophy 1 isoform D OS=Drosophila melanogaster OX=7227 GN=Opa1 PE=1 SV=1 |
| Q9VAM6 | CDGSH iron-sulfur domain-containing protein 2 homolog OS=Drosophila melanogaster OX=7227 GN=Cisd2 PE=2 SV=1 |
| Q9VZF1 | GH07444p OS=Drosophila melanogaster OX=7227 GN=Dmel\CG1309 PE=1 SV=1 |
| Q8T6I0 | EH domain containing protein OS=Drosophila melanogaster OX=7227 GN=Past1 PE=1 SV=1 |
| X2J4W8 | Kruppel homolog 2 isoform C OS=Drosophila melanogaster OX=7227 GN=Kr-h2 PE=1 SV=1 |
| Q9V9U2 | FI19428p1 OS=Drosophila melanogaster OX=7227 GN=salt PE=2 SV=2 |
| Q6NP72 | GEO09659p1 OS=Drosophila melanogaster OX=7227 GN=Dmel\CG13220 PE=1 SV=1 |
| Q9VGM2 | Fatty acid binding protein isoform B OS=Drosophila melanogaster OX=7227 GN=fabp PE=1 SV=1 |
| A1Z729 | Uncharacterized protein OS=Drosophila melanogaster OX=7227 GN=Dmel\CG2064 PE=1 SV=1 |
| M9PJN8 | Glyceraldehyde-3-phosphate dehydrogenase OS=Drosophila melanogaster OX=7227 GN=Gapdh2 PE=3 SV=1 |
| O97418 | EG:152A3.7 protein OS=Drosophila melanogaster OX=7227 GN=ND-B14.5A PE=1 SV=1 |
| G4LTX1 | GH14535p2 OS=Drosophila melanogaster OX=7227 GN=Pmi PE=1 SV=1 |
| Q9W4H6 | Pyruvate dehydrogenase E1 component subunit alpha OS=Drosophila melanogaster OX=7227 GN=Pdha PE=1 SV=2 |
| A0A0B4KGN2 | MICOS complex subunit MIC60 OS=Drosophila melanogaster OX=7227 GN=Mitofilin PE=3 SV=1 |
| Q9V4E7 | Transporter OS=Drosophila melanogaster OX=7227 GN=Gat PE=1 SV=4 |
| M9NEX3 | Uncharacterized protein isoform D OS=Drosophila melanogaster OX=7227 GN=BcDNA:RH45308 PE=1 SV=1 |
| Q8SYJ2 | GEO09626p1 OS=Drosophila melanogaster OX=7227 GN=ND-MLRQ PE=1 SV=1 |
| O18333 | GH01619p OS=Drosophila melanogaster OX=7227 GN=Rab2 PE=1 SV=1 |
| Q6IHT7 | HDC01001 OS=Drosophila melanogaster OX=7227 GN=Sfp26Ad PE=4 SV=1 |
| Q9V3Y4 | LD43650p OS=Drosophila melanogaster OX=7227 GN=Mtch PE=1 SV=1 |
| Q01604 | Phosphoglycerate kinase OS=Drosophila melanogaster OX=7227 GN=Pgk PE=2 SV=2 |
| Q9U915 | Adenylate kinase OS=Drosophila melanogaster OX=7227 GN=Adk2 PE=1 SV=1 |
| Q9V3L7 | GEO04710p1 OS=Drosophila melanogaster OX=7227 GN=NP15.6 PE=1 SV=1 |
| Q8IQH0 | FI12817p OS=Drosophila melanogaster OX=7227 GN=Nrx-IV PE=1 SV=2 |
| Q9VES6 | Fatty acyl-CoA reductase OS=Drosophila melanogaster OX=7227 GN=Dmel\CG17560 PE=3 SV=2 |
| Q9XYZ9 | Glutathione S transferase E12 isoform A OS=Drosophila melanogaster OX=7227 GN=GstE12 PE=1 SV=1 |
| Q8IMJ0 | Uncharacterized protein isoform A OS=Drosophila melanogaster OX=7227 GN=anon-EST:Posey81 PE=1 SV=1 |
| C8VV60 | FI03659p OS=Drosophila melanogaster OX=7227 GN=CG10616-RA PE=1 SV=1 |
| Q960M4 | Peroxiredoxin OS=Drosophila melanogaster OX=7227 GN=Prx5 PE=1 SV=1 |
| Q9VF20 | Heavy metal tolerance factor 1 OS=Drosophila melanogaster OX=7227 GN=Hmt-1 PE=1 SV=1 |
| Q9VD58 | GH26270p OS=Drosophila melanogaster OX=7227 GN=Idh3b PE=1 SV=1 |
| Q8SZ28 | RE21371p OS=Drosophila melanogaster OX=7227 GN=CG3884 PE=1 SV=1 |
| P18432 | Myosin regulatory light chain 2 OS=Drosophila melanogaster OX=7227 GN=Mlc2 PE=1 SV=2 |
| A0A0B4LGB7 | Calcium-transporting ATPase OS=Drosophila melanogaster OX=7227 GN=SERCA PE=1 SV=1 |
| P15007 | Enolase OS=Drosophila melanogaster OX=7227 GN=Eno PE=1 SV=2 |
| Q9VV72 | Multiple inositol polyphosphate phosphatase 1 OS=Drosophila melanogaster OX=7227 GN=Mipp1 PE=1 SV=2 |
| Q7K5K3 | Pyruvate dehydrogenase E1 component subunit beta OS=Drosophila melanogaster OX=7227 GN=Pdhb PE=1 SV=1 |
| Q59E30 | Failed axon connections isoform C OS=Drosophila melanogaster OX=7227 GN=fax PE=1 SV=1 |
| O77062 | Amino acid transporter OS=Drosophila melanogaster OX=7227 GN=Eaat1 PE=1 SV=1 |
| Q7K221 | Glutamate oxaloacetate transaminase 1 isoform A OS=Drosophila melanogaster OX=7227 GN=Got1 PE=1 SV=1 |
| Q94915 | Rhythmically expressed gene 2 protein OS=Drosophila melanogaster OX=7227 GN=Reg-2 PE=2 SV=1 |
| Q9V7D2 | V-type proton ATPase subunit D 1 OS=Drosophila melanogaster OX=7227 GN=Vha36-1 PE=2 SV=1 |
| Q9VVL7 | Dihydrolipoyl dehydrogenase OS=Drosophila melanogaster OX=7227 GN=E3 PE=1 SV=1 |
| P48148 | Ras-like GTP-binding protein Rho1 OS=Drosophila melanogaster OX=7227 GN=Rho1 PE=1 SV=1 |
| Q00637 | Superoxide dismutase [Mn] mitochondrial OS=Drosophila melanogaster OX=7227 GN=Sod2 PE=2 SV=3 |
| P53501 | Actin-57B OS=Drosophila melanogaster OX=7227 GN=Act57B PE=1 SV=1 |
| Q23982 | Ejaculatory bulb-specific protein 2 OS=Drosophila melanogaster OX=7227 GN=EbpII PE=2 SV=2 |
| Q9VTP4 | 60S ribosomal protein L10a-2 OS=Drosophila melanogaster OX=7227 GN=RpL10Ab PE=1 SV=2 |
| Q95TZ7 | GH19182p OS=Drosophila melanogaster OX=7227 GN=Zasp66 PE=1 SV=1 |
| Q9I7R1 | NADH-cytochrome b5 reductase OS=Drosophila melanogaster OX=7227 GN=Dmel\CG5946 PE=1 SV=2 |
| X2JEM4 | GEO07185p1 OS=Drosophila melanogaster OX=7227 GN=RpS28b PE=2 SV=1 |
| Q9W2X6 | ATP synthase delta subunit isoform A OS=Drosophila melanogaster OX=7227 GN=ATPsyndelta PE=1 SV=1 |
| Q9VNW6 | Delta-1-pyrroline-5-carboxylate synthase OS=Drosophila melanogaster OX=7227 GN=Dmel\CG7470 PE=1 SV=1 |
| X2JE06 | GEO07624p1 OS=Drosophila melanogaster OX=7227 GN=RpL24 PE=1 SV=1 |
| Q9VUY9 | Phosphoglucomutase OS=Drosophila melanogaster OX=7227 GN=Pgm PE=1 SV=1 |
| Q9W1I7 | GH07506p OS=Drosophila melanogaster OX=7227 GN=Dmel\CG5554 PE=1 SV=1 |
| A0A0B4JCW4 | Succinate--CoA ligase [ADP-forming] subunit beta mitochondrial OS=Drosophila melanogaster OX=7227 GN=ScsbetaA PE=1 SV=1 |
| Q961R9 | GH09241p OS=Drosophila melanogaster OX=7227 GN=CT19169 PE=1 SV=1 |
| Q9Y115 | UNC93-like protein OS=Drosophila melanogaster OX=7227 GN=CG4928 PE=2 SV=1 |
| P91927 | Mitochondrial proton/calcium exchanger protein OS=Drosophila melanogaster OX=7227 GN=Letm1 PE=2 SV=2 |
| O77266 | Uncharacterized protein isoform A OS=Drosophila melanogaster OX=7227 GN=Dmel\CG4199 PE=1 SV=3 |
| Q9VKP2 | GH26960p OS=Drosophila melanogaster OX=7227 GN=Porin2 PE=2 SV=1 |
| Q9VI17 | SCP domain-containing protein OS=Drosophila melanogaster OX=7227 GN=Dmel\CG42564 PE=4 SV=2 |
| Q9VFQ9 | Dipeptidase B isoform A OS=Drosophila melanogaster OX=7227 GN=Dip-B PE=1 SV=2 |
| P41043 | Glutathione S-transferase S1 OS=Drosophila melanogaster OX=7227 GN=GstS1 PE=1 SV=2 |
| A1ZB79 | Uncharacterized protein isoform M OS=Drosophila melanogaster OX=7227 GN=Dmel\CG5174 PE=1 SV=2 |
| Q7JRC3 | Epoxide hydrolase OS=Drosophila melanogaster OX=7227 GN=Jheh1 PE=2 SV=1 |
| Q9VIL5 | GEO08953p1 OS=Drosophila melanogaster OX=7227 GN=Dmel\CG17472 PE=2 SV=1 |
| Q94522 | Succinate--CoA ligase [ADP/GDP-forming] subunit alpha mitochondrial OS=Drosophila melanogaster OX=7227 GN=Scsalpha1 PE=2 SV=3 |
| Q9VEB1 | IP09655p OS=Drosophila melanogaster OX=7227 GN=Mdh2 PE=1 SV=1 |
| Q9VLQ7 | Serpin 28F OS=Drosophila melanogaster OX=7227 GN=Spn28F PE=3 SV=2 |
| Q9VD61 | Sulfhydryl oxidase OS=Drosophila melanogaster OX=7227 GN=Qsox2 PE=2 SV=1 |
| Q9VM12 | MIP26555p1 OS=Drosophila melanogaster OX=7227 GN=Dmel\CG5958 PE=1 SV=1 |
| Q7K084 | Odorant-binding protein 44a isoform A OS=Drosophila melanogaster OX=7227 GN=Obp44a PE=1 SV=1 |
| Q9W4W8 | Paraplegin isoform A OS=Drosophila melanogaster OX=7227 GN=Spg7 PE=1 SV=1 |
| Q94920 | Voltage-dependent anion-selective channel OS=Drosophila melanogaster OX=7227 GN=porin PE=1 SV=3 |
| Q9VQB4 | LD06553p OS=Drosophila melanogaster OX=7227 GN=Dmel\CG3609 PE=1 SV=1 |
| Q9VVA4 | GH26789p OS=Drosophila melanogaster OX=7227 GN=GS PE=1 SV=2 |
| Q9VIH9 | GEO04151p1 OS=Drosophila melanogaster OX=7227 GN=BcDNA:GH07967 PE=2 SV=1 |
| D1Z3A2 | ATP synthase-coupling factor 6 mitochondrial OS=Drosophila melanogaster OX=7227 GN=ATPsynCF6 PE=1 SV=1 |
| Q9VAX7 | Tubulin beta chain OS=Drosophila melanogaster OX=7227 GN=betaTub97EF PE=1 SV=3 |
| Q9VXZ0 | GEO07190p1 OS=Drosophila melanogaster OX=7227 GN=ND-B18 PE=1 SV=1 |
| Q7K5N8 | GH01592p OS=Drosophila melanogaster OX=7227 GN=BEST:GH19547 PE=1 SV=1 |
| B5RIM9 | Glycerol-3-phosphate dehydrogenase [NAD(+)] OS=Drosophila melanogaster OX=7227 GN=Gpdh1 PE=1 SV=1 |
| Q9VAN7 | Phosphoglycerate mutase OS=Drosophila melanogaster OX=7227 GN=Pglym78 PE=1 SV=2 |
| Q9VZL1 | LP07226p OS=Drosophila melanogaster OX=7227 GN=mge PE=1 SV=1 |
| Q9V427 | Innexin inx2 OS=Drosophila melanogaster OX=7227 GN=Inx2 PE=1 SV=1 |
| P45594 | Cofilin/actin-depolymerizing factor homolog OS=Drosophila melanogaster OX=7227 GN=tsr PE=1 SV=1 |
| X2J979 | Synaptotagmin 1 isoform H OS=Drosophila melanogaster OX=7227 GN=Syt1 PE=4 SV=1 |
| Q9W306 | GEO08256p1 OS=Drosophila melanogaster OX=7227 GN=CT27394 PE=1 SV=1 |
| Q8SXY6 | Transmembrane emp24 domain-containing protein bai OS=Drosophila melanogaster OX=7227 GN=bai PE=2 SV=1 |
| P08879 | Nucleoside diphosphate kinase OS=Drosophila melanogaster OX=7227 GN=awd PE=1 SV=3 |
| P14318 | Muscle-specific protein 20 OS=Drosophila melanogaster OX=7227 GN=Mp20 PE=2 SV=2 |
| Q9VHP0 | ATP-dependent RNA helicase bel OS=Drosophila melanogaster OX=7227 GN=bel PE=1 SV=1 |
| Q9VPJ9 | Uncharacterized protein isoform B OS=Drosophila melanogaster OX=7227 GN=CK02656 PE=1 SV=2 |
| O62619 | Pyruvate kinase OS=Drosophila melanogaster OX=7227 GN=PyK PE=2 SV=2 |
| P32748 | Dihydroorotate dehydrogenase (quinone) mitochondrial OS=Drosophila melanogaster OX=7227 GN=Dhod PE=2 SV=2 |
| P17704 | 40S ribosomal protein S17 OS=Drosophila melanogaster OX=7227 GN=RpS17 PE=1 SV=2 |
| A1ZBJ2 | Uncharacterized protein isoform B OS=Drosophila melanogaster OX=7227 GN=cg7461 PE=1 SV=2 |
| Q9W1H6 | GH04238p OS=Drosophila melanogaster OX=7227 GN=Dmel\CG5597 PE=1 SV=1 |
| Q9V3W2 | GM23292p OS=Drosophila melanogaster OX=7227 GN=ND-B17 PE=1 SV=1 |
| Q9V3E7 | LD24793p OS=Drosophila melanogaster OX=7227 GN=Ref1 PE=1 SV=1 |
| P15372 | Phosrestin-2 OS=Drosophila melanogaster OX=7227 GN=Arr1 PE=1 SV=1 |
| M9NGK3 | Terribly reduced optic lobes isoform AL OS=Drosophila melanogaster OX=7227 GN=trol PE=1 SV=2 |
| Q9VF27 | GM02062p OS=Drosophila melanogaster OX=7227 GN=ND-23 PE=1 SV=1 |
| E1JH55 | Aquaporin OS=Drosophila melanogaster OX=7227 GN=AQP PE=3 SV=1 |
| A1ZA47 | PDZ and LIM domain protein Zasp OS=Drosophila melanogaster OX=7227 GN=Zasp52 PE=1 SV=2 |
| Q9VX69 | FI01450p OS=Drosophila melanogaster OX=7227 GN=Dmel\CG5162 PE=1 SV=1 |
| P48588 | 40S ribosomal protein S25 OS=Drosophila melanogaster OX=7227 GN=RpS25 PE=1 SV=3 |
| P06603 | Tubulin alpha-1 chain OS=Drosophila melanogaster OX=7227 GN=alphaTub84B PE=1 SV=1 |
| M9PJQ5 | Wings up A isoform K OS=Drosophila melanogaster OX=7227 GN=wupA PE=1 SV=1 |
| Q8SXR1 | Neural lazarillo isoform A OS=Drosophila melanogaster OX=7227 GN=NLaz PE=1 SV=1 |
| Q9VIE8 | Aconitate hydratase mitochondrial OS=Drosophila melanogaster OX=7227 GN=mAcon1 PE=1 SV=2 |
| Q8T4C4 | ATP synthase subunit beta OS=Drosophila melanogaster OX=7227 GN=ATPsynbetaL PE=2 SV=1 |
| R9PY16 | Uncharacterized protein OS=Drosophila melanogaster OX=7227 GN=Ubi-p5E5 PE=4 SV=1 |
| Q95U34 | GH11113p OS=Drosophila melanogaster OX=7227 GN=Galk PE=1 SV=1 |
| Q9VQA3 | Uncharacterized protein isoform A OS=Drosophila melanogaster OX=7227 GN=anon-SAGE:Wang-110 PE=4 SV=1 |
| P00334 | Alcohol dehydrogenase OS=Drosophila melanogaster OX=7227 GN=Adh PE=1 SV=2 |
