# Supplementary material for: The dysregulated Pink1- Drosophila mitochondrial proteome is partially corrected with exercise
Source: Aging (Albany NY). 2021 Jun 1;13(11):14709–28. doi: 10.18632/aging.203128 (PMC8221352; doi:10.18632/aging.203128)
Supplement: Supplementary Table 2 [file aging-13-203128-s004.docx]

**Supplementary Table 2.Label-free MS identified 105 proteins with different abundance when comparing non-exercised *Pink1^-^* and wildtype flies.**

| **Protein** | **Heat-map Row** | **Database identifier** | **Mean Diff.** | **95.00% CI of diff.** | **Adjusted *p-*value** |  | **Pink1-/- n.e relative to WT n.e** |
| --- | --- | --- | --- | --- | --- | --- | --- |
| NADPH--cytochrome P450 reductase | 4 | Q27597 | 11.33 | 2.379 to 20.29 | 0.0063 | ** | ↑ |
| GH20802p | 5 | Q9W3L4 | 14 | 5.045 to 22.95 | 0.0003 | *** | ↑ |
| Transferrin | 7 | Q9VWV6 | 9.667 | 0.7120 to 18.62 | 0.0284 | * | ↑ |
| Fatty acyl-CoA reductase | 10 | Q9VLJ7 | 16 | 7.045 to 24.95 | <0.0001 | **** | ↑ |
| Heat shock protein 60A | 11 | O02649 | 9.333 | 0.3787 to 18.29 | 0.0372 | * | ↑ |
| FI01422p | 12 | A1Z6V5 | 13 | 4.045 to 21.95 | 0.0011 | ** | ↑ |
| Chitinase-like protein Idgf4 | 15 | Q9W303 | 9.667 | 0.7120 to 18.62 | 0.0284 | * | ↑ |
| Enoyl-CoA hydratase short chain 1 isoform A | 16 | Q7JR58 | 9.667 | 0.7120 to 18.62 | 0.0284 | * | ↑ |
| LD30155p | 27 | Q9VN21 | 10.67 | 1.712 to 19.62 | 0.0119 | * | ↑ |
| FI09602p | 31 | Q9VVU1 | 11.33 | 2.379 to 20.29 | 0.0063 | ** | ↑ |
| Reticulon-like protein | 32 | Q9VMV9 | 14.33 | 5.379 to 23.29 | 0.0002 | *** | ↑ |
| Sc2 | 34 | Q9VZL3 | 12.33 | 3.379 to 21.29 | 0.0023 | ** | ↑ |
| CG3835-RA | 38 | Q7K511 | 9.333 | 0.3787 to 18.29 | 0.0372 | * | ↑ |
| Ferritin | 39 | Q7KRU8 | 9 | 0.04537 to 17.95 | 0.0483 | * | ↑ |
| Sideroflexin-1-3 | 41 | Q9VN13 | 10 | 1.045 to 18.95 | 0.0215 | * | ↑ |
| LP10861p | 44 | Q9W0M4 | 13 | 4.045 to 21.95 | 0.0011 | ** | ↑ |
| EG:BACR7A4.14 protein | 48 | Q9U1L2 | 12.67 | 3.712 to 21.62 | 0.0016 | ** | ↑ |
| A-kinase anchor protein 200 | 50 | Q9VLL3 | 11 | 2.045 to 19.95 | 0.0087 | ** | ↑ |
| RE08669p | 51 | Q9V9W3 | 12 | 3.045 to 20.95 | 0.0032 | ** | ↑ |
| Cytochrome b5 | 52 | Q9V4N3 | 9.333 | 0.3787 to 18.29 | 0.0372 | * | ↑ |
| Cytochrome P450 4e2 | 53 | Q27606 | 11.67 | 2.712 to 20.62 | 0.0046 | ** | ↑ |
| Sphingosine-1-phosphate lyase | 61 | Q9V7Y2 | 12.67 | 3.712 to 21.62 | 0.0016 | ** | ↑ |
| Rm62 isoform H | 64 | E1JJ68 | 15 | 6.045 to 23.95 | 0.0001 | *** | ↑ |
| 40S ribosomal protein S14b | 65 | C0HKA1 | 13 | 4.045 to 21.95 | 0.0011 | ** | ↑ |
| LD12946p | 66 | Q9VXM4 | 10.67 | 1.712 to 19.62 | 0.0119 | * | ↑ |
| 60S ribosomal protein L5 | 67 | Q9W5R8 | 9.667 | 0.7120 to 18.62 | 0.0284 | * | ↑ |
| Probable cytochrome P450 9f2 | 68 | Q9VG82 | 10.33 | 1.379 to 19.29 | 0.0161 | * | ↑ |
| HDC00331 | 70 | Q6IHY5 | -10.33 | -19.29 to -1.379 | 0.0161 | * | ↓ |
| Sodium/potassium-transporting ATPase subunit beta-1 | 72 | Q24046 | -9 | -17.95 to -0.04537 | 0.0483 | * | ↓ |
| Receptor of activated protein kinase C 1 isoform C | 73 | M9PCC1 | 10.67 | 1.712 to 19.62 | 0.0119 | * | ↑ |
| GH22187p | 74 | Q7YTZ4 | 9 | 0.04537 to 17.95 | 0.0483 | * | ↑ |
| Probable cytochrome P450 28a5 | 75 | Q9V419 | 25.67 | 16.71 to 34.62 | <0.0001 | **** | ↑ |
| RE29555p | 79 | Q9VC18 | 9.333 | 0.3787 to 18.29 | 0.0372 | * | ↑ |
| GH11762p | 80 | Q9VY05 | 9 | 0.04537 to 17.95 | 0.0483 | * | ↑ |
| Polyadenylate-binding protein | 81 | P21187 | 13 | 4.045 to 21.95 | 0.0011 | ** | ↑ |
| Cytochrome P450 9b2 | 82 | Q9V4I1 | 14.67 | 5.712 to 23.62 | 0.0002 | *** | ↑ |
| Dolichyl-diphosphooligosaccharide--protein glycosyltransferase subunit 2 | 83 | A0A0B4LFR4 | 11.67 | 2.712 to 20.62 | 0.0046 | ** | ↑ |
| Fatty acyl-CoA reductase | 84 | Q9VG87 | 11.33 | 2.379 to 20.29 | 0.0063 | ** | ↑ |
| LD30122p | 86 | Q9W4N8 | 14.33 | 5.379 to 23.29 | 0.0002 | *** | ↑ |
| 40S ribosomal protein S23 | 88 | Q8T3U2 | 9.667 | 0.7120 to 18.62 | 0.0284 | * | ↑ |
| Uncharacterized protein isoform A | 89 | Q86BQ3 | 12.67 | 3.712 to 21.62 | 0.0016 | ** | ↑ |
| Poly(U)-specific endoribonuclease homolog | 90 | Q9VZ49 | 10 | 1.045 to 18.95 | 0.0215 | * | ↑ |
| Zipper isoform H | 91 | A0A0B4JD95 | 15.67 | 6.712 to 24.62 | <0.0001 | **** | ↑ |
| Glutamine synthetase | 93 | E1JHQ1 | 9 | 0.04537 to 17.95 | 0.0483 | * | ↑ |
| RH34413p | 95 | Q9VU35 | 11 | 2.045 to 19.95 | 0.0087 | ** | ↑ |
| Ribosomal protein L11 isoform B | 97 | A0A0B4LGZ5 | 9.333 | 0.3787 to 18.29 | 0.0372 | * | ↑ |
| ATP binding cassette subfamily B member 7 isoform B | 98 | Q7KVB1 | 17.67 | 8.712 to 26.62 | <0.0001 | **** | ↑ |
| GM05240p | 99 | Q9VCC6 | 11.67 | 2.712 to 20.62 | 0.0046 | ** | ↑ |
| BcDNA.GH10229 | 104 | Q9Y114 | 15.67 | 6.712 to 24.62 | <0.0001 | **** | ↑ |
| Probable transaldolase | 106 | Q9W1G0 | 12 | 3.045 to 20.95 | 0.0032 | ** | ↑ |
| Heat shock protein 22 | 113 | P02515 | 19.33 | 10.38 to 28.29 | <0.0001 | **** | ↑ |
| CG6459 protein | 114 | Q7JXC4 | 13.67 | 4.712 to 22.62 | 0.0005 | *** | ↑ |
| Phosphatidate cytidylyltransferase | 116 | X2JC55 | 12 | 3.045 to 20.95 | 0.0032 | ** | ↑ |
| 60S ribosomal protein L9 | 118 | P50882 | 10.67 | 1.712 to 19.62 | 0.0119 | * | ↑ |
| NADH dehydrogenase (Ubiquinone) 75 kDa subunit isoform B | 119 | A4V449 | -9.333 | -18.29 to -0.3787 | 0.0372 | * | ↓ |
| AT07710p | 120 | Q9VHW0 | 9.667 | 0.7120 to 18.62 | 0.0284 | * | ↑ |
| 40S ribosomal protein S7 | 123 | Q8IMI7 | 9.333 | 0.3787 to 18.29 | 0.0372 | * | ↑ |
| LD24105p | 127 | Q9W3N9 | 12 | 3.045 to 20.95 | 0.0032 | ** | ↑ |
| Fatty acid synthase 3 | 130 | Q7PLB8 | 10 | 1.045 to 18.95 | 0.0215 | * | ↑ |
| Putative fatty acyl-CoA reductase CG8306 | 133 | Q960W6 | 9 | 0.04537 to 17.95 | 0.0483 | * | ↑ |
| Fatty acid synthase 2 isoform A | 135 | Q9VQL6 | 10 | 1.045 to 18.95 | 0.0215 | * | ↑ |
| Microsomal glutathione S-transferase-like isoform A | 137 | Q8SY19 | 9.333 | 0.3787 to 18.29 | 0.0372 | * | ↑ |
| Annexin | 140 | A0A0B4KH34 | 11.33 | 2.379 to 20.29 | 0.0063 | ** | ↑ |
| Fatty acyl-CoA reductase | 145 | Q9VES7 | 17.33 | 8.379 to 26.29 | <0.0001 | **** | ↑ |
| Probable cytochrome P450 6a23 | 147 | Q9V771 | -11 | -19.95 to -2.045 | 0.0087 | ** | ↓ |
| Imaginal disk growth factor 6 | 149 | Q23997 | 9.667 | 0.7120 to 18.62 | 0.0284 | * | ↑ |
| Calnexin 99A isoform A | 150 | Q9VAL7 | 9 | 0.04537 to 17.95 | 0.0483 | * | ↑ |
| C-1-tetrahydrofolate synthase cytoplasmic | 151 | O96553 | 10.67 | 1.712 to 19.62 | 0.0119 | * | ↑ |
| Peptidyl-prolyl cis-trans isomerase | 157 | Q9W227 | 9.333 | 0.3787 to 18.29 | 0.0372 | * | ↑ |
| GH01077p | 161 | Q9VFF0 | -9.333 | -18.29 to -0.3787 | 0.0372 | * | ↓ |
| Epoxide hydrolase | 162 | Q7KB18 | 9.333 | 0.3787 to 18.29 | 0.0372 | * | ↑ |
| Catalase | 167 | P17336 | 9 | 0.04537 to 17.95 | 0.0483 | * | ↑ |
| HL01062p | 168 | Q9VU17 | 10 | 1.045 to 18.95 | 0.0215 | * | ↑ |
| Tropomyosin 2 isoform E | 169 | A0A0B4KHJ9 | -10.67 | -19.62 to -1.712 | 0.0119 | * | ↓ |
| Maltase A1 | 179 | P07190 | 9.667 | 0.7120 to 18.62 | 0.0284 | * | ↑ |
| GH02075p | 219 | Q9VPR1 | 16.67 | 7.712 to 25.62 | <0.0001 | **** | ↑ |
| Eukaryotic translation initiation factor 3 subunit C | 226 | A0A0B4LFL2 | 17 | 8.045 to 25.95 | <0.0001 | **** | ↑ |
| 60S acidic ribosomal protein P0 | 230 | M9PG76 | 9.333 | 0.3787 to 18.29 | 0.0372 | * | ↑ |
| RE74312p | 232 | Q9VD29 | 10 | 1.045 to 18.95 | 0.0215 | * | ↑ |
| Uncharacterized protein isoform B | 240 | M9MSJ3 | 10.67 | 1.712 to 19.62 | 0.0119 | * | ↑ |
| UDP-glucuronosyltransferase | 292 | Q9W2J4 | -13.33 | -22.29 to -4.379 | 0.0008 | *** | ↓ |
| Actin indirect flight muscle | 315 | P83967 | -20.67 | -29.62 to -11.71 | <0.0001 | **** | ↓ |
| UDP-glucose:glycoprotein glucosyltransferase | 329 | Q09332 | 13.67 | 4.712 to 22.62 | 0.0005 | *** | ↑ |
| Translocon-associated protein subunit beta | 334 | Q9VUZ0 | 10.33 | 1.379 to 19.29 | 0.0161 | * | ↑ |
| GM14617p | 352 | Q9V9T5 | 17 | 8.045 to 25.95 | <0.0001 | **** | ↑ |
| Levy isoform A | 353 | Q9W1N3 | -18.33 | -27.29 to -9.379 | <0.0001 | **** | ↓ |
| Extended synaptotagmin-like protein 2 isoform D | 357 | A0A0B4KGU9 | 21.33 | 12.38 to 30.29 | <0.0001 | **** | ↑ |
| RH59310p | 381 | Q9W3N1 | 3.333 | -5.621 to 12.29 | 0.774 | ns | ↑ |
| 40S ribosomal protein S18 | 383 | P41094 | 9.667 | 0.7120 to 18.62 | 0.0284 | * | ↑ |
| GEO07462p1 | 387 | M9MRC9 | 15 | 6.045 to 23.95 | 0.0001 | *** | ↑ |
| Uncharacterized protein isoform B | 390 | X2JEA2 | 12.33 | 3.379 to 21.29 | 0.0023 | ** | ↑ |
| Aminomethyltransferase | 392 | Q9VKR4 | 17.67 | 8.712 to 26.62 | <0.0001 | **** | ↑ |
| Uncharacterized protein | 395 | Q8IPP8 | 10 | 1.045 to 18.95 | 0.0215 | * | ↑ |
| HDC01001 | 417 | Q6IHT7 | 14.67 | 5.712 to 23.62 | 0.0002 | *** | ↑ |
| Fatty acyl-CoA reductase | 423 | Q9VES6 | 9.333 | 0.3787 to 18.29 | 0.0372 | * | ↑ |
| FI03659p | 426 | C8VV60 | 15 | 6.045 to 23.95 | 0.0001 | *** | ↑ |
| Heavy metal tolerance factor 1 | 428 | Q9VF20 | 9.667 | 0.7120 to 18.62 | 0.0284 | * | ↑ |
| 60S ribosomal protein L10a-2 | 446 | Q9VTP4 | 9.333 | 0.3787 to 18.29 | 0.0372 | * | ↑ |
| NADH-cytochrome b5 reductase | 448 | Q9I7R1 | 11.67 | 2.712 to 20.62 | 0.0046 | ** | ↑ |
| GEO07185p1 | 449 | X2JEM4 | 9 | 0.04537 to 17.95 | 0.0483 | * | ↑ |
| GH07506p | 454 | Q9W1I7 | 10.33 | 1.379 to 19.29 | 0.0161 | * | ↑ |
| Mitochondrial proton/calcium exchanger protein | 458 | P91927 | 9.667 | 0.7120 to 18.62 | 0.0284 | * | ↑ |
| Sulfhydryl oxidase | 470 | Q9VD61 | -14.67 | -23.62 to -5.712 | 0.0002 | *** | ↓ |
| Paraplegin isoform A | 473 | Q9W4W8 | 9.667 | 0.7120 to 18.62 | 0.0284 | * | ↑ |
| GH26789p | 476 | Q9VVA4 | 13 | 4.045 to 21.95 | 0.0011 | ** | ↑ |
