# Supplementary material for: The dysregulated Pink1- Drosophila mitochondrial proteome is partially corrected with exercise
Source: Aging (Albany NY). 2021 Jun 1;13(11):14709–28. doi: 10.18632/aging.203128 (PMC8221352; doi:10.18632/aging.203128)
Supplement: Supplementary Table 3 [file aging-13-203128-s005.docx]

**Supplementary Table 3. Label-free MS identified 57 proteins with altered abundance between exercised and non-exercised *Pink1^-^* flies, 55 of which had decreased abundance.**

| **Protein** | **Heat-map Row** | **Database identifier** | **Mean Diff.** | **95.00% CI of diff.** | **Adjusted *p-*value** |  | **Change /w exercise** |
| --- | --- | --- | --- | --- | --- | --- | --- |
| Peroxisomal multifunctional enzyme type 2 isoform B | 26 | X2JFD6 | 9.333 | 0.3787 to 18.29 | 0.0372 | * | ↓ |
| Sc2 | 34 | Q9VZL3 | 10.67 | 1.712 to 19.62 | 0.0119 | * | ↓ |
| EG:BACR7A4.14 protein | 48 | Q9U1L2 | 11 | 2.045 to 19.95 | 0.0087 | ** | ↓ |
| Ribosomal protein S10b isoform D | 60 | M9NEQ9 | 9.333 | 0.3787 to 18.29 | 0.0372 | * | ↓ |
| Rm62 isoform H | 64 | E1JJ68 | 11 | 2.045 to 19.95 | 0.0087 | ** | ↓ |
| Receptor of activated protein kinase C 1 isoform C | 73 | M9PCC1 | 11.33 | 2.379 to 20.29 | 0.0063 | ** | ↓ |
| Probable cytochrome P450 28a5 | 75 | Q9V419 | 23.67 | 14.71 to 32.62 | <0.0001 | **** | ↓ |
| RE29555p | 79 | Q9VC18 | 9 | 0.04537 to 17.95 | 0.0483 | * | ↓ |
| Cytochrome P450 9b2 | 82 | Q9V4I1 | 10.67 | 1.712 to 19.62 | 0.0119 | * | ↓ |
| GH23390p | 85 | Q95SI7 | 11.33 | 2.379 to 20.29 | 0.0063 | ** | ↓ |
| Zipper isoform H | 91 | A0A0B4JD95 | 12.33 | 3.379 to 21.29 | 0.0023 | ** | ↓ |
| Probable cytochrome P450 12a4 mitochondrial | 92 | Q9VE00 | 9.333 | 0.3787 to 18.29 | 0.0372 | * | ↓ |
| Ribosomal protein L11 isoform B | 97 | A0A0B4LGZ5 | 10.33 | 1.379 to 19.29 | 0.0161 | * | ↓ |
| ATP binding cassette subfamily B member 7 isoform B | 98 | Q7KVB1 | 14.33 | 5.379 to 23.29 | 0.0002 | *** | ↓ |
| GM05240p | 99 | Q9VCC6 | 10.67 | 1.712 to 19.62 | 0.0119 | * | ↓ |
| 60S ribosomal protein L28 | 102 | Q9VZS5 | 11 | 2.045 to 19.95 | 0.0087 | ** | ↓ |
| BcDNA.GH10229 | 104 | Q9Y114 | 10.67 | 1.712 to 19.62 | 0.0119 | * | ↓ |
| GH26015p | 107 | Q7K3N4 | 15.33 | 6.379 to 24.29 | <0.0001 | **** | ↓ |
| CathD isoform A | 115 | Q7K485 | 17.33 | 8.379 to 26.29 | <0.0001 | **** | ↓ |
| LD24105p | 127 | Q9W3N9 | 11.67 | 2.712 to 20.62 | 0.0046 | ** | ↓ |
| Annexin | 140 | A0A0B4KH34 | 10 | 1.045 to 18.95 | 0.0215 | * | ↓ |
| Imaginal disk growth factor 6 | 149 | Q23997 | 12 | 3.045 to 20.95 | 0.0032 | ** | ↓ |
| Acetyl-CoA carboxylase isoform B | 163 | Q7JV23 | 9.667 | 0.7120 to 18.62 | 0.0284 | * | ↓ |
| GH02075p | 219 | Q9VPR1 | 9.667 | 0.7120 to 18.62 | 0.0284 | * | ↓ |
| Eukaryotic translation initiation factor 3 subunit C | 226 | A0A0B4LFL2 | 14.33 | 5.379 to 23.29 | 0.0002 | *** | ↓ |
| 60S acidic ribosomal protein P0 | 230 | M9PG76 | 9.333 | 0.3787 to 18.29 | 0.0372 | * | ↓ |
| RE74312p | 232 | Q9VD29 | 12.33 | 3.379 to 21.29 | 0.0023 | ** | ↓ |
| Aminopeptidase | 237 | Q8IN25 | 9.667 | 0.7120 to 18.62 | 0.0284 | * | ↓ |
| FI02856p | 270 | Q9VII1 | 9.333 | 0.3787 to 18.29 | 0.0372 | * | ↓ |
| LD31742p | 276 | Q7JQH9 | 14.33 | 5.379 to 23.29 | 0.0002 | *** | ↓ |
| Neither inactivation nor afterpotential protein C | 282 | P10676 | 10.33 | 1.379 to 19.29 | 0.0161 | * | ↓ |
| Glutathione S transferase E9 | 286 | Q7K8X7 | 11 | 2.045 to 19.95 | 0.0087 | ** | ↓ |
| Hsc70Cb isoform A | 296 | Q9VUC1 | 9.333 | 0.3787 to 18.29 | 0.0372 | * | ↓ |
| OCIA domain-containing protein 1 | 308 | Q9W1X9 | -9.667 | -18.62 to -0.7120 | 0.0284 | * | ↑ |
| UDP-glucose:glycoprotein glucosyltransferase | 329 | Q09332 | 12 | 3.045 to 20.95 | 0.0032 | ** | ↓ |
| Translocon-associated protein subunit beta | 334 | Q9VUZ0 | 10.33 | 1.379 to 19.29 | 0.0161 | * | ↓ |
| 6-phosphogluconate dehydrogenase decarboxylating | 341 | P41572 | 9 | 0.04537 to 17.95 | 0.0483 | * | ↓ |
| GM14617p | 352 | Q9V9T5 | 11 | 2.045 to 19.95 | 0.0087 | ** | ↓ |
| Extended synaptotagmin-like protein 2 isoform D | 357 | A0A0B4KGU9 | 18.67 | 9.712 to 27.62 | <0.0001 | **** | ↓ |
| GEO07753p1 | 362 | Q9VY92 | 9.333 | 0.3787 to 18.29 | 0.0372 | * | ↓ |
| 40S ribosomal protein S24 | 364 | Q9W229 | 10.33 | 1.379 to 19.29 | 0.0161 | * | ↓ |
| 40S ribosomal protein S18 | 383 | P41094 | 10.33 | 1.379 to 19.29 | 0.0161 | * | ↓ |
| GEO07462p1 | 387 | M9MRC9 | 17.33 | 8.379 to 26.29 | <0.0001 | **** | ↓ |
| Aminomethyltransferase | 392 | Q9VKR4 | 19 | 10.05 to 27.95 | <0.0001 | **** | ↓ |
| FI19428p1 | 404 | Q9V9U2 | 13.67 | 4.712 to 22.62 | 0.0005 | *** | ↓ |
| GH14535p2 | 410 | G4LTX1 | 10.33 | 1.379 to 19.29 | 0.0161 | * | ↓ |
| Uncharacterized protein isoform D | 414 | M9NEX3 | 9.333 | 0.3787 to 18.29 | 0.0372 | * | ↓ |
| HDC01001 | 417 | Q6IHT7 | 10.67 | 1.712 to 19.62 | 0.0119 | * | ↓ |
| Uncharacterized protein isoform A | 425 | Q8IMJ0 | 13 | 4.045 to 21.95 | 0.0011 | ** | ↓ |
| Peroxiredoxin | 427 | Q960M4 | 9.333 | 0.3787 to 18.29 | 0.0372 | * | ↓ |
| 60S ribosomal protein L10a-2 | 446 | Q9VTP4 | 15 | 6.045 to 23.95 | 0.0001 | *** | ↓ |
| GEO07185p1 | 449 | X2JEM4 | 13.33 | 4.379 to 22.29 | 0.0008 | *** | ↓ |
| Epoxide hydrolase | 465 | Q7JRC3 | 12 | 3.045 to 20.95 | 0.0032 | ** | ↓ |
| Paraplegin isoform A | 473 | Q9W4W8 | 10 | 1.045 to 18.95 | 0.0215 | * | ↓ |
| GH26789p | 476 | Q9VVA4 | 14.67 | 5.712 to 23.62 | 0.0002 | *** | ↓ |
| Uncharacterized protein isoform B | 493 | Q9VPJ9 | 12.33 | 3.379 to 21.29 | 0.0023 | ** | ↓ |
| Dihydroorotate dehydrogenase (quinone) mitochondrial | 495 | P32748 | -15.67 | -24.62 to -6.712 | <0.0001 | **** | ↑ |
