# Supplementary material for: The dysregulated Pink1- Drosophila mitochondrial proteome is partially corrected with exercise
Source: Aging (Albany NY). 2021 Jun 1;13(11):14709–28. doi: 10.18632/aging.203128 (PMC8221352; doi:10.18632/aging.203128)
Supplement: Supplementary Table 4 [file aging-13-203128-s006.docx]

**Supplementary Table 4. Label-free MS identified 56 proteins with different abundance when comparing exercised *Pink1^-^* and exercised WT flies.**

| **Protein** | **Heat-map Row** | **Database identifier** | **Mean Diff.** | **95.00% CI of diff.** | **Adjusted *p-*value** |  | ***Pink1-/-* e relative to WT e** |
| --- | --- | --- | --- | --- | --- | --- | --- |
| Endoplasmic reticulum chaperone BiP | 2 | P29844 | 10 | 1.045 to 18.95 | 0.0215 | * | ↑ |
| GH20802p | 5 | Q9W3L4 | 12 | 3.045 to 20.95 | 0.0032 | ** | ↑ |
| Cytochrome c oxidase subunit 4 isoform A | 6 | Q9VIQ8 | -12.33 | -21.29 to -3.379 | 0.0023 | ** | ↓ |
| Cyclope isoform A | 8 | Q9VMS1 | -10 | -18.95 to -1.045 | 0.0215 | * | ↓ |
| FI01422p | 12 | A1Z6V5 | 11.33 | 2.379 to 20.29 | 0.0063 | ** | ↑ |
| LD47962p | 13 | Q9V4E0 | -13.67 | -22.62 to -4.712 | 0.0005 | *** | ↓ |
| NADH dehydrogenase [ubiquinone] flavoprotein 1 mitochondrial | 14 | Q9VMI3 | -11 | -19.95 to -2.045 | 0.0087 | ** | ↓ |
| Enoyl-CoA hydratase short chain 1 isoform A | 16 | Q7JR58 | 9 | 0.04537 to 17.95 | 0.0483 | * | ↑ |
| NADH dehydrogenase (Ubiquinone) B16.6 subunit isoform A | 19 | Q9W402 | -9.333 | -18.29 to -0.3787 | 0.0372 | * | ↓ |
| Flightin isoform B | 22 | M9PD14 | -17.33 | -26.29 to -8.379 | <0.0001 | **** | ↓ |
| Cytochrome b-c1 complex subunit 7 | 23 | Q9VXI6 | -12.33 | -21.29 to -3.379 | 0.0023 | ** | ↓ |
| NADH dehydrogenase [ubiquinone] 1 alpha subcomplex subunit 12 | 29 | Q9VQD7 | -11 | -19.95 to -2.045 | 0.0087 | ** | ↓ |
| FI09602p | 31 | Q9VVU1 | 12 | 3.045 to 20.95 | 0.0032 | ** | ↑ |
| Cytochrome c oxidase subunit 2 | 35 | P00408 | -9.667 | -18.62 to -0.7120 | 0.0284 | * | ↓ |
| Troponin I | 36 | P36188 | -11 | -19.95 to -2.045 | 0.0087 | ** | ↓ |
| Glutamine synthetase | 37 | X2JJG8 | 9.667 | 0.7120 to 18.62 | 0.0284 | * | ↑ |
| LP10861p | 44 | Q9W0M4 | 10 | 1.045 to 18.95 | 0.0215 | * | ↑ |
| NADH dehydrogenase (Ubiquinone) 18 kDa subunit | 47 | Q9VWI0 | -13 | -21.95 to -4.045 | 0.0011 | ** | ↓ |
| RE08669p | 51 | Q9V9W3 | 9.333 | 0.3787 to 18.29 | 0.0372 | * | ↑ |
| Cytochrome P450 4e2 | 53 | Q27606 | 9.333 | 0.3787 to 18.29 | 0.0372 | * | ↑ |
| Cytochrome c oxidase subunit | 54 | Q8IQW2 | -9.667 | -18.62 to -0.7120 | 0.0284 | * | ↓ |
| HDC00331 | 70 | Q6IHY5 | -16 | -24.95 to -7.045 | <0.0001 | **** | ↓ |
| SD02021p | 71 | Q9VRJ4 | -14 | -22.95 to -5.045 | 0.0003 | *** | ↓ |
| Protein disulfide-isomerase A6 homolog | 76 | Q9V438 | 10.67 | 1.712 to 19.62 | 0.0119 | * | ↑ |
| Probable methylmalonate-semialdehyde dehydrogenase [acylating] mitochondrial | 77 | Q7KW39 | 12.33 | 3.379 to 21.29 | 0.0023 | ** | ↑ |
| NADH dehydrogenase [ubiquinone] 1 subunit C2 | 78 | Q9VQM2 | -10.67 | -19.62 to -1.712 | 0.0119 | * | ↓ |
| Polyadenylate-binding protein | 81 | P21187 | 9 | 0.04537 to 17.95 | 0.0483 | * | ↑ |
| Poly(U)-specific endoribonuclease homolog | 90 | Q9VZ49 | 11 | 2.045 to 19.95 | 0.0087 | ** | ↑ |
| Probable cytochrome P450 12a4 mitochondrial | 92 | Q9VE00 | -11.33 | -20.29 to -2.379 | 0.0063 | ** | ↓ |
| Glutamine synthetase | 93 | E1JHQ1 | 9.667 | 0.7120 to 18.62 | 0.0284 | * | ↑ |
| Putative ferric-chelate reductase 1 homolog | 105 | Q8MSU3 | -9.333 | -18.29 to -0.3787 | 0.0372 | * | ↓ |
| Flotillin-1 | 111 | O61491 | 13.33 | 4.379 to 22.29 | 0.0008 | *** | ↑ |
| Heat shock protein 22 | 113 | P02515 | 13 | 4.045 to 21.95 | 0.0011 | ** | ↑ |
| Phosphatidate cytidylyltransferase | 116 | X2JC55 | 9.667 | 0.7120 to 18.62 | 0.0284 | * | ↑ |
| V-type proton ATPase subunit d 1 | 117 | Q9W4P5 | -14 | -22.95 to -5.045 | 0.0003 | *** | ↓ |
| Fatty acyl-CoA reductase | 145 | Q9VES7 | 10 | 1.045 to 18.95 | 0.0215 | * | ↑ |
| Probable cytochrome P450 6a23 | 147 | Q9V771 | -12.33 | -21.29 to -3.379 | 0.0023 | ** | ↓ |
| C-1-tetrahydrofolate synthase cytoplasmic | 151 | O96553 | 9 | 0.04537 to 17.95 | 0.0483 | * | ↑ |
| NADH dehydrogenase [ubiquinone] 1 beta subcomplex subunit 8 mitochondrial | 156 | Q9W3X7 | -9 | -17.95 to -0.04537 | 0.0483 | * | ↓ |
| Cytochrome c oxidase subunit 5A mitochondrial | 193 | Q94514 | -10.67 | -19.62 to -1.712 | 0.0119 | * | ↓ |
| GEO11443p1 | 211 | Q500Y7 | -13.67 | -22.62 to -4.712 | 0.0005 | *** | ↓ |
| Uncharacterized protein isoform B | 216 | Q9W2H8 | -11 | -19.95 to -2.045 | 0.0087 | ** | ↓ |
| Coiled-coil-helix-coiled-coil-helix domain containing 3 | 244 | Q9VA18 | -20.33 | -29.29 to -11.38 | <0.0001 | **** | ↓ |
| NADH dehydrogenase (Ubiquinone) B14.7 subunit | 246 | Q7JYH3 | -10 | -18.95 to -1.045 | 0.0215 | * | ↓ |
| Accessory gland protein 36DE isoform B | 261 | X2J8Y6 | 10 | 1.045 to 18.95 | 0.0215 | * | ↑ |
| LD31742p | 276 | Q7JQH9 | -11.33 | -20.29 to -2.379 | 0.0063 | ** | ↓ |
| Cytochrome b-c1 complex subunit 9 | 311 | Q9XY35 | -9 | -17.95 to -0.04537 | 0.0483 | * | ↓ |
| NADH dehydrogenase (Ubiquinone) B14 subunit isoform A | 343 | Q7JZK1 | -13 | -21.95 to -4.045 | 0.0011 | ** | ↓ |
| Levy isoform A | 353 | Q9W1N3 | -14.33 | -23.29 to -5.379 | 0.0002 | *** | ↓ |
| 40S ribosomal protein S18 | 383 | P41094 | -13.67 | -22.62 to -4.712 | 0.0005 | *** | ↓ |
| GH14535p2 | 410 | G4LTX1 | -15 | -23.95 to -6.045 | 0.0001 | *** | ↓ |
| GEO09626p1 | 415 | Q8SYJ2 | -17 | -25.95 to -8.045 | <0.0001 | **** | ↓ |
| RE21371p | 430 | Q8SZ28 | 10.67 | 1.712 to 19.62 | 0.0119 | * | ↑ |
| Glutamate oxaloacetate transaminase 1 isoform A | 438 | Q7K221 | -9.667 | -18.62 to -0.7120 | 0.0284 | * | ↓ |
| GEO08256p1 | 488 | Q9W306 | 9.333 | 0.3787 to 18.29 | 0.0372 | * | ↑ |
| Dihydroorotate dehydrogenase (quinone) mitochondrial | 495 | P32748 | 13 | 4.045 to 21.95 | 0.0011 | ** | ↑ |
