# Supplementary material for: The dysregulated Pink1- Drosophila mitochondrial proteome is partially corrected with exercise
Source: Aging (Albany NY). 2021 Jun 1;13(11):14709–28. doi: 10.18632/aging.203128 (PMC8221352; doi:10.18632/aging.203128)
Supplement: Supplementary Table 5 [file aging-13-203128-s007.docx]

**Supplementary Table 5. Label-free MS identified 55 proteins with different abundance when comparing exercised *Pink1^-^* and non-exercised WT flies.**

| **Protein** | **Heat-map Row** | **Database identifier** | **Mean Diff.** | **95.00% CI of diff.** | **Adjusted *p-*value** |  | ***Pink1-/-* e relative to WT n.e** |
| --- | --- | --- | --- | --- | --- | --- | --- |
| Succinate dehydrogenase [ubiquinone] flavoprotein subunit mitochondrial | 1 | Q94523 | -9 | -17.95 to -0.04537 | 0.0483 | * | ↓ |
| GH20802p | 5 | Q9W3L4 | 11.33 | 2.379 to 20.29 | 0.0063 | ** | ↑ |
| Cytochrome c oxidase subunit 4 isoform A | 6 | Q9VIQ8 | -9 | -17.95 to -0.04537 | 0.0483 | * | ↓ |
| FI01422p | 12 | A1Z6V5 | 11.33 | 2.379 to 20.29 | 0.0063 | ** | ↑ |
| LD47962p | 13 | Q9V4E0 | -9 | -17.95 to -0.04537 | 0.0483 | * | ↓ |
| Flightin isoform B | 22 | M9PD14 | -9 | -17.95 to -0.04537 | 0.0483 | * | ↓ |
| FI09602p | 31 | Q9VVU1 | 11.67 | 2.712 to 20.62 | 0.0046 | ** | ↑ |
| Glutamine synthetase | 37 | X2JJG8 | 9.333 | 0.3787 to 18.29 | 0.0372 | * | ↑ |
| LP10861p | 44 | Q9W0M4 | 9.667 | 0.7120 to 18.62 | 0.0284 | * | ↑ |
| RE08669p | 51 | Q9V9W3 | 10 | 1.045 to 18.95 | 0.0215 | * | ↑ |
| Myosin alkali light chain 1 isoform D | 63 | A0A0B4LHS1 | -12.33 | -21.29 to -3.379 | 0.0023 | ** | ↓ |
| HDC00331 | 70 | Q6IHY5 | -9.667 | -18.62 to -0.7120 | 0.0284 | * | ↓ |
| Probable methylmalonate-semialdehyde dehydrogenase [acylating] mitochondrial | 77 | Q7KW39 | 10.33 | 1.379 to 19.29 | 0.0161 | * | ↑ |
| Polyadenylate-binding protein | 81 | P21187 | 9.333 | 0.3787 to 18.29 | 0.0372 | * | ↑ |
| Fatty acyl-CoA reductase | 84 | Q9VG87 | 9 | 0.04537 to 17.95 | 0.0483 | * | ↑ |
| GH23390p | 85 | Q95SI7 | -9.333 | -18.29 to -0.3787 | 0.0372 | * | ↓ |
| Poly(U)-specific endoribonuclease homolog | 90 | Q9VZ49 | 11.67 | 2.712 to 20.62 | 0.0046 | ** | ↑ |
| Probable cytochrome P450 12a4 mitochondrial | 92 | Q9VE00 | -12.33 | -21.29 to -3.379 | 0.0023 | ** | ↓ |
| Glycogen [starch] synthase | 94 | A0A0B4KHJ5 | -10 | -18.95 to -1.045 | 0.0215 | * | ↓ |
| GH26015p | 107 | Q7K3N4 | -11 | -19.95 to -2.045 | 0.0087 | ** | ↓ |
| Flotillin-1 | 111 | O61491 | 11.33 | 2.379 to 20.29 | 0.0063 | ** | ↑ |
| Heat shock protein 22 | 113 | P02515 | 13.67 | 4.712 to 22.62 | 0.0005 | *** | ↑ |
| CathD isoform A | 115 | Q7K485 | -10 | -18.95 to -1.045 | 0.0215 | * | ↓ |
| Phosphatidate cytidylyltransferase | 116 | X2JC55 | 10 | 1.045 to 18.95 | 0.0215 | * | ↑ |
| V-type proton ATPase subunit d 1 | 117 | Q9W4P5 | -10 | -18.95 to -1.045 | 0.0215 | * | ↓ |
| ATP-dependent 6-phosphofructokinase | 128 | A0A0B4K7L1 | -9.333 | -18.29 to -0.3787 | 0.0372 | * | ↓ |
| Probable cytochrome P450 6d5 | 132 | Q9VFP1 | 11.33 | 2.379 to 20.29 | 0.0063 | ** | ↑ |
| Fatty acyl-CoA reductase | 145 | Q9VES7 | 10.33 | 1.379 to 19.29 | 0.0161 | * | ↑ |
| Probable cytochrome P450 6a23 | 147 | Q9V771 | -14 | -22.95 to -5.045 | 0.0003 | *** | ↓ |
| Tropomyosin 2 isoform E | 169 | A0A0B4KHJ9 | -10.67 | -19.62 to -1.712 | 0.0119 | * | ↓ |
| BcDNA.GH10614 | 210 | Q9Y112 | -10 | -18.95 to -1.045 | 0.0215 | * | ↓ |
| GEO11443p1 | 211 | Q500Y7 | -9.333 | -18.29 to -0.3787 | 0.0372 | * | ↓ |
| Uncharacterized protein isoform B | 216 | Q9W2H8 | -13.67 | -22.62 to -4.712 | 0.0005 | *** | ↓ |
| Aminopeptidase | 237 | Q8IN25 | -9 | -17.95 to -0.04537 | 0.0483 | * | ↓ |
| UDP-glucuronosyltransferase | 248 | Q9VGT8 | -10 | -18.95 to -1.045 | 0.0215 | * | ↓ |
| UDP-glucuronosyltransferase | 292 | Q9W2J4 | -15.67 | -24.62 to -6.712 | <0.0001 | **** | ↓ |
| GH25683p | 302 | Q9VXG9 | -10 | -18.95 to -1.045 | 0.0215 | * | ↓ |
| OCIA domain-containing protein 1 | 308 | Q9W1X9 | 10.67 | 1.712 to 19.62 | 0.0119 | * | ↑ |
| Cytochrome b-c1 complex subunit 9 | 311 | Q9XY35 | -10 | -18.95 to -1.045 | 0.0215 | * | ↓ |
| Actin indirect flight muscle | 315 | P83967 | -19.67 | -28.62 to -10.71 | <0.0001 | **** | ↓ |
| Mitochondrial pyruvate carrier | 327 | A0A0B4KHD3 | -12.33 | -21.29 to -3.379 | 0.0023 | ** | ↓ |
| 6-phosphogluconate dehydrogenase decarboxylating | 341 | P41572 | -9 | -17.95 to -0.04537 | 0.0483 | * | ↓ |
| NADH dehydrogenase (Ubiquinone) B14 subunit isoform A | 343 | Q7JZK1 | -9.333 | -18.29 to -0.3787 | 0.0372 | * | ↓ |
| Levy isoform A | 353 | Q9W1N3 | -18.67 | -27.62 to -9.712 | <0.0001 | **** | ↓ |
| GEO07753p1 | 362 | Q9VY92 | -9 | -17.95 to -0.04537 | 0.0483 | * | ↓ |
| GH14252p | 388 | Q95U15 | -10.67 | -19.62 to -1.712 | 0.0119 | * | ↓ |
| Seminal fluid protein 24Bb | 398 | B4ZJ91 | -9.333 | -18.29 to -0.3787 | 0.0372 | * | ↓ |
| FI03659p | 426 | C8VV60 | 9.333 | 0.3787 to 18.29 | 0.0372 | * | ↑ |
| Peroxiredoxin | 427 | Q960M4 | -12.67 | -21.62 to -3.712 | 0.0016 | ** | ↓ |
| Enolase | 433 | P15007 | -10.33 | -19.29 to -1.379 | 0.0161 | * | ↓ |
| Multiple inositol polyphosphate phosphatase 1 | 434 | Q9VV72 | -12 | -20.95 to -3.045 | 0.0032 | ** | ↓ |
| Sulfhydryl oxidase | 470 | Q9VD61 | -16.33 | -25.29 to -7.379 | <0.0001 | **** | ↓ |
| MIP26555p1 | 471 | Q9VM12 | -9.667 | -18.62 to -0.7120 | 0.0284 | * | ↓ |
| LP07226p | 484 | Q9VZL1 | 9.667 | 0.7120 to 18.62 | 0.0284 | * | ↑ |
| Dihydroorotate dehydrogenase (quinone) mitochondrial | 495 | P32748 | 15.67 | 6.712 to 24.62 | <0.0001 | **** | ↑ |
